# From Ecological Threats to Monitoring Tools: Multi-Contaminant Profiles in *Silurus glanis* and *Procambarus clarkii* for Pollution Tracking and Preliminary Food/Feed Safety Assessment

**DOI:** 10.3390/jox16030109

**Published:** 2026-06-09

**Authors:** Sara Glorio Patrucco, Roberta Giugliano, Alessandra Griglione, Giorgia Zicarelli, Camilla Mossotto, Leo Costa, Giuseppe Esposito, Alice Gabetti, Serena Anselmi, Tecla Bentivoglio, Barbara Vivaldi, Valentina Ciccotelli, Bruno Aimone, Marino Prearo, Damià Barceló, Monia Renzi, Stefania Squadrone, Paolo Pastorino

**Affiliations:** 1 Istituto Zooprofilattico Sperimentale del Piemonte, Liguria e Valle d’Aosta, 10154 Torino, Italy; sara.gloriopatrucco@izsplv.it (S.G.P.); alessandra.griglione@izsplv.it (A.G.); giorgia.zicarelli@izsplv.it (G.Z.); camilla.mossotto@izsplv.it (C.M.); leo.costa@izsplv.it (L.C.); giuseppe.esposito@izsplv.it (G.E.); alice.gabetti@izsplv.it (A.G.); marino.prearo@izsplv.it (M.P.); stefania.squadrone@izsplv.it (S.S.); 2 Istituto Zooprofilattico Sperimentale del Piemonte, Liguria e Valle d’Aosta, 16129 Genova, Italy; roberta.giugliano@izsplv.it (R.G.); barbara.vivaldi@izsplv.it (B.V.); valentina.ciccotelli@izsplv.it (V.C.); 3 Bioscience Research Center, 58015 Orbetello, Italy; serena.anselmi@bsrc.it (S.A.); tecla.bentivoglio@bsrc.it (T.B.); 4 Ente di Gestione delle Aree Protette delle Alpi Cozie, 10050 Salbertrand, Italy; aimone@alpicozie.eu; 5 Chemistry and Physics Department, University of Almeria, 04120 Almería, Spain; damiab@ual.es; 6 Sino-Spain Joint Laboratory for Agricultural Environment Emerging Contaminants of Zhejiang Province, Zhejiang A&F University, Hangzhou 311300, China; 7 Department of Life Sciences, University of Trieste, 34127 Trieste, Italy; mrenzi@units.it

**Keywords:** bioindicators, invasive alien species, microplastics, NDL-PCBs, pesticides, REEs, trace elements

## Abstract

Invasive alien species (IAS) such as *Silurus glanis* and *Procambarus clarkii* represent major ecological threats but may also serve as effective bioindicators of environmental contamination; therefore, this study aimed to evaluate their potential for multi-contaminant monitoring and assess their suitability as alternative feed and food resources within a circular economy framework. Multi-contaminant profiles were investigated in *S. glanis* and *P. clarkii* from Avigliana Lakes (NW Italy), analyzing trace elements, rare earth elements (REEs), and organic contaminants in fish muscle, and microplastics (MPs) in intestinal tracts. In *S. glanis*, total trace element concentrations and ΣREEs were markedly higher in Small Lake than in Great Lake, with ΣREEs reaching 0.445 and 0.056 mg/kg w.w., respectively. Mean concentrations of the regulated elements in Great Lake were 0.017 mg/kg w.w. (As), 0.003 mg/kg w.w. (Cd), and 0.16 mg/kg w.w. (Pb), increasing in Small Lake to 0.19, 0.03, and 1.86 mg/kg w.w., respectively. In *P. clarkii*, contamination levels were lower, with ΣREEs averaging 0.074 mg/kg w.w. and mean concentrations of As, Cd, and Pb of 0.25, 0.006, and 0.21 mg/kg w.w., respectively. Organic contaminants, including polycyclic aromatic hydrocarbons (PAHs), non-dioxin-like polychlorinated biphenyls (NDL-PCBs), and pesticides, were generally below limits of quantification. MPs were detected in 100% of specimens, with mean concentrations of 4.2 ± 2.15 and 4.4 ± 2.70 MPs per intestinal tract in *S. glanis* (Great and Small Lake, respectively) and 2.7 ± 2.39 MPs/intestinal tract in *P. clarkii*. Permutational multivariate analysis of variance (PERMANOVA) indicated significant site-related differences in *S. glanis* and species-related differences between *S. glanis* and *P. clarkii* within Great Lake. Most regulated contaminants were below applicable EU thresholds; however, Pb in *S. glanis* from Small Lake exceeded the maximum level established for fish muscle intended for human consumption.

## 1. Introduction

The increasing spread of invasive alien species (IAS) represents a significant threat to freshwater biodiversity and ecosystem integrity [1]. IAS are recognized as one of the main drivers of global biodiversity loss, increasing extinction risk for numerous critically endangered species through competitive interactions and altering ecosystem functioning via predation, disease transmission, and habitat modification [2].

Among invasive freshwater species, the European catfish (*Silurus glanis* Linnaeus, 1758) and the red swamp crayfish (*Procambarus clarkii* Girard, 1852) are of increasing concern in several countries, particularly in southern and central Europe, where favorable environmental conditions facilitate their establishment and spread [3,4].

*Silurus glanis* is considered a highly problematic IAS due to its position as a top predator, its opportunistic feeding behavior, aggressive activity during the spawning period, and the high predation pressure it exerts on native fish communities [5]. Native to Eastern Europe and Western Asia, the species has been widely introduced beyond its natural range, mainly in Western Europe for sport fishing and aquaculture purposes. Established populations have also been reported in North Africa and Brazil [6,7].

Similarly, *P. clarkii* has become one of the most widespread invasive crayfish species worldwide due to its high ecological plasticity, rapid reproductive rate, and tolerance to a wide range of environmental conditions. Its presence can substantially alter aquatic habitats and competitively displace native species, in some cases leading to local extinctions [8]. Native to Southern United States and Northern Mexico, the red swamp crayfish has been introduced to numerous regions globally and is now considered an invasive species in many areas, particularly across Europe and South Africa [9].

The eradication and management of IAS in European freshwater systems are guided by the EU Invasive Alien Species Regulation (Regulation (EU) No 1143/2014) [10], which entered into force in January 2015 and requires Member States to implement measures for prevention, early detection, and rapid eradication of species of Union concern. In Italy, this regulation has been transposed into national legislation through Italian Legislative Decree No. 230/2017 [11]. Among the listed species, the red swamp crayfish is included in the Union list, requiring Member States to adopt targeted eradication and control measures to mitigate its ecological impacts.

While *S. glanis* is not yet on the Union list, it is recognized as a high-risk invasive predator in several European rivers and lakes and is the focus of multiple eradication initiatives, including the LIFE Predator project [5,12], aimed at reducing its pressure on native freshwater biodiversity. To prevent further spread, some Italian regions have also implemented regional containment plans, particularly within protected areas [13,14,15].

Given the extensive eradication efforts, both *S. glanis* and *P. clarkii* provide a unique opportunity for environmental monitoring. Recent studies have suggested using IAS as alternative bioindicators for environmental chemistry and pollution monitoring (trace elements, persistent pollutants, emerging contaminants, etc.) [2]. Unlike many native species, which are often vulnerable, declining, or legally protected due to conservation concerns, IAS are generally abundant, widespread, and naturalized for many years, thus reflecting the environmental conditions of the ecosystems they inhabit. These traits make sampling easier and minimize ethical and conservation issues associated with monitoring native species [2].

Furthermore, within a circular economy perspective, IAS are increasingly being considered as potential alternative feed and food sources [16,17]. However, the assessment of contaminant levels is essential to ensure both animal feed and human food safety, as these organisms can accumulate harmful substances [18]. In this context, analyzing contaminants in IAS can provide valuable insights not only into environmental pollution but also into the safety of these species as food or feed matrices. Despite their potential as bioindicators and potential feed/food sources, studies focusing on *S. glanis* and *P. clarkii* remain limited.

This study is based on the hypothesis that these IAS can effectively accumulate a wide range of environmental contaminants in a site- and species-dependent manner, with higher concentrations expected in more impacted sites, thereby reflecting local pollution levels and providing relevant information regarding their safety as potential feed and food sources.

Accordingly, the present study aims to investigate the bioaccumulation of multiple classes of contaminants, including trace elements and rare earth elements, persistent organic contaminants (polycyclic aromatic hydrocarbons, non-dioxin-like polychlorinated biphenyls, and pesticides), and emerging contaminants (microplastics), in *S. glanis* and *P. clarkii* in a protected area of Piedmont (Avigliana Lakes Nature Park, Northwest Italy). Muscle tissues were analyzed for trace elements, rare earth elements, and organic contaminants, whereas intestinal tracts were analyzed for microplastics. The Avigliana Lakes were selected because they are located within a protected area where these invasive species are established and subject to mandatory control and eradication measures. Their removal provides a unique opportunity to evaluate contaminant burdens while also assessing their potential destination and safe use as alternative feed or food resources within a circular economy framework.

## 2. Materials and Methods

### 2.1. Study Area

**Figure 1:**
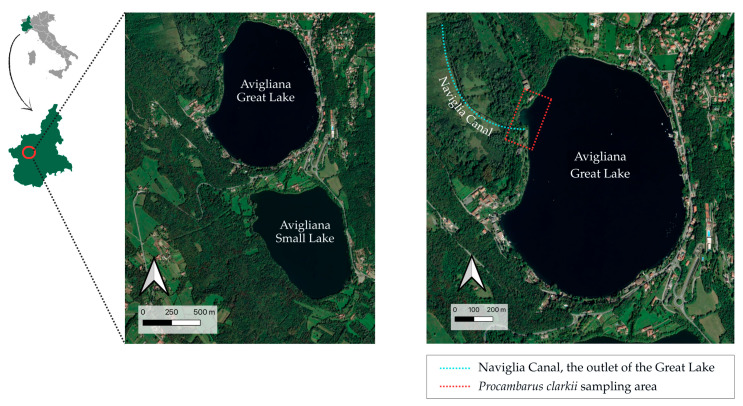
Study area: Avigliana Great and Small Lakes, Piedmont Region, NW Italy. The sampling sites of *P. clarkii*, as indicated by the red dotted rectangle, included a section of the shoreline of the Great Lake and a portion of the Naviglia Canal. Sampling sites for *S. glanis* are not indicated on the map since the longline transects spanned the entire length of the lakes’ perimeter and electrofishing sessions were carried out along the entire shoreline as well.

The study area includes two lakes, Avigliana Great Lake and Avigliana Small Lake, located within the Avigliana Lakes Nature Park in the lower Susa Valley (Piedmont, Northwestern Italy) (Figure 1). The lakes are of glacial origin and formed during the retreat of Alpine glaciers. The climate in the area is temperate, with precipitation concentrated in the autumn and spring months. The two lakes are connected by the Rio Meana, a short watercourse approximately 400 m long, while the outflow from the Great Lake, known as the Naviglia Canal, passes through the Mareschi Marsh and eventually flows into the Dora Riparia. Small Lake (approximately 60 ha; 356 m a.s.l., maximum depth 12 m) is characterized by a relatively natural environment, surrounded by woodlands, meadows, and reed beds that provide habitat for fish and bird species. It receives part of its water from a drained peat bog, the Torbiera di Trana. The shores of the Great Lake (approximately 90 ha; 352 m a.s.l., maximum depth 27 m) have been significantly altered by human activities. Since 2012, the Avigliana Lakes Nature Park has been part of the protected areas network of the Cottian Alps. The site is included in the Natura 2000 and is designated both as a Site of Community Importance (SCI; IT1110007) under the EU Habitats Directive and as a Special Protection Area (SPA) under the EU Birds Directive [19,20]. Although the study area is part of a protected area, it is affected by surrounding human activities: industrial areas, agricultural land, and urban centers, all of which can be sources of pollution. Both lakes are popular destinations for tourists and anglers.

### 2.2. Silurus glanis Sampling

A total of 15 individuals of *S. glanis* were captured from Great Lake (total length: mean 76 cm, range 35–143 cm; weight: mean 3.98 kg, range 0.28–14.46 kg), and 15 individuals from Small Lake (total length: mean 66 cm, range 23–100 cm; weight: mean 2.25 kg, range 0.07–5.70 kg) using a combination of electrofishing and longlines. Longlines, a technique particularly suitable for lacustrine environments and commonly employed in invasive species control programs, were placed along the entire shoreline of both lakes. The electrofishing surveys were carried out along the shoreline as well. The sampling campaign was carried out from April to September 2025. All fishing operations were carried out by personnel appointed by the Park Authority (Ente di Gestione delle Aree Protette delle Alpi Cozie), under specific authorizations (see Institutional Review Board Statement).

To ensure the integrity of subsequent analyses, all specimens were transported in refrigerated containers from sampling area to laboratory. Upon arrival, each individual was weighed and measured in length. Muscle and intestinal tissues were collected from every specimen within 24 h of capture and stored at −20 °C until further contaminant analysis.

### 2.3. Procambarus clarkii Sampling

A total of 40 individuals of *P. clarkii* were captured along the northwestern shores of the Great Lake and partly in the Naviglia Canal (Figure 1) (total length: mean 9 cm, range 7–11 cm; weight: mean 23.37 g, range 10.04–36.27 g). Most crayfish are concentrated in this area due to the presence of favorable habitats, including non-artificial banks and submerged vegetation, which create an ideal environment for burrow digging. Other areas of the Great Lake are characterized by anthropogenic modifications (*e.g.,* artificial banks) and unfavorable depths, which, combined with a lack of vegetation, limit the available habitat for *P. clarkii.* Sampling in Small Lake was not conducted, as *P. clarkii* has not yet been detected in this lake [19]. The specimen collection was carried out from April to September 2025. Crayfish sampling was conducted using cylindrical traps with double funnel-shaped entrances, baited with canned cat food (100 g net weight), in accordance with national guidelines for the control of invasive crayfish species [21,22]. The canned cat food was used solely as an olfactory attractant. Traps (approximately 88 cm in length and 16–37 cm in diameter) were deployed along the shores of the Great Lake at intervals of about 50 m, at a depth of 0.5 m, and retrieved after 24 h.

Captured crayfishes were temporarily placed in refrigerated containers to induce anesthesia via exposure to low temperatures. This method slows metabolic activity, limits movement, and reduces aggressive behavior. The specimens were subsequently transported to the laboratory, where they were euthanized and disposed of in compliance with current regulations [22,23]. Each captured specimen was weighed, and muscle and intestinal tissues were collected and stored at −20 °C until contaminant analysis.

### 2.4. Contaminants Analysis

Muscle samples of *S. glanis* (N = 15 Great Lake, N = 15 Small Lake) and *P. clarkii* (N = 40 Great Lake) were analyzed for the detection of trace elements, rare earth elements (REEs) and organic contaminants (polycyclic aromatic hydrocarbons-PAHs), non-dioxin-like polychlorinated biphenyls-NDL-PCBs, and pesticides.

Intestinal tracts of *S. glanis* (N = 15 Great Lake, N = 15 Small Lake) and *P. clarkii* (N = 40 Great Lake) were used to detect microplastics (MPs).

#### 2.4.1. Detection of Trace Elements and Rare Earth Elements (REEs)

The detection of 21 trace elements (Ag, As, Ba, Bi, Cd, Co, Cr, Cs, Cu, Ga, In, Mn, Mo, Ni, Pb, Rb, Se, Sr, Tl, U, V) and the 16 REEs—including 14 lanthanides (Ce, Dy, Er, Eu, Gd, Ho, La, Lu, Nd, Pr, Sm, Tb, Tm, and Yb) as well as Sc and Y—was conducted following previously described protocol [24].

Briefly, samples (1.5–2.0 g of frozen muscle) were subjected to wet digestion using high-purity acids and oxidants (7 mL of HNO_3_, 70% *v*/*v*, and 1.5 mL of H_2_O_2_, 30% *v*/*v*) in a microwave system (ETHOS 1, Milestone, Shelton, CT, USA). After digestion, ultrapure water was added to reach a final weight of 50 g (Arium 611VF, Sartorius Stedim Italy S.p.A., Antella—Bagno a Ripoli, FI, Italy). Multi-elemental analysis was carried out using inductively coupled plasma mass spectrometry (ICP-MS, Agilent 7800, Santa Clara, CA, USA), with external calibration curves and internal standards. Blank values were subtracted only for trace elements, not for REEs. The limit of quantification (LOQ) for each element was 0.001 mg kg^−1^, and the limit of detection (LOD) was 0.0003 mg kg^−1^. Concentrations were expressed on a wet-weight (w.w.) basis. Certified reference materials, recoveries, precision/RSD, blank values, and internal standards are reported in Supplementary Material (Text S1).

##### Chondrite-Normalized REE Patterns

To enable comparison among samples and remove the natural abundance-related trend of REEs, concentrations were normalized to chondritic reference values according to McDonough and Sun [25]. Chondrite-normalized concentrations (REE_N_) were calculated as:

(1) $$REE_{N}=\frac{REE_{sample}}{REE_{chondrite}}$$

using the following reference values (in µg/g): La = 0.234, Ce = 0.613, Pr = 0.092, Nd = 0.457, Sm = 0.153, Eu = 0.058, Gd = 0.199, Tb = 0.036, Dy = 0.249, Ho = 0.054, Er = 0.165, Tm = 0.025, Yb = 0.161, and Lu = 0.024. To assess REE fractionation and potential redox-related processes, Ce and Eu anomalies were calculated as:

(2) $$Ce/Ce^{*}=\frac{Ce_{N}}{\sqrt{La_{N}\cdot Pr_{N}}}$$

(3) $$Eu/Eu^{*}=\frac{Eu_{N}}{\sqrt{Sm_{N}\cdot Gd_{N}}}$$

REE patterns were visualized using a logarithmic scale. Mean normalized REE profiles were calculated for each species and sampling site.

#### 2.4.2. Detection of Organic Contaminants

##### Detection of Polycyclic Aromatic Hydrocarbons (PAHs)

The detection of benzo[a]anthracene-B[a]A, chrysene-Chr, benzo[b]fluoranthene-B[b]F, and benzo[a]pyrene-B[a]P was performed following the method previously described [26].

Samples were extracted using a QuEChERS-based procedure. Briefly, 5 mL of ultrapure water were added to the sample and vortex-mixed to ensure complete wetting of the matrix. Subsequently, 10 mL of acetonitrile were added, and the mixture was shaken using a vibratory shaker.

A QuEChERS extraction salt mixture, consisting of 4 g MgSO_4_ and 1 g NaCl (manually weighed), was then added. The samples were vigorously shaken on a rocking platform for 5 min and centrifuged at 3900 rpm for 10 min to achieve phase separation.

Approximately 8 mL of the organic supernatant were transferred into 15 mL screw-cap tubes and placed in a freezer at −70 to −90 °C for at least 1 h. Before completing thawing, the extracts were centrifuged at 3900 rpm for 5 min to remove any precipitated material.

An aliquot of 6 mL of the organic phase was transferred into tubes containing QuEChERS d-SPE clean-up sorbents (900 mg MgSO_4_ and 150 mg PSA). The tubes were capped and shaken vigorously on a rocking platform for 5 min, followed by centrifugation at 3900 rpm for 10 min.

From the resulting supernatant, 1 mL was transferred into Eppendorf tubes and centrifuged at 9000 rpm for 10 min using a microcentrifuge. The final extracts were transferred into appropriately labelled vials. A blank sample was prepared using approximately 1 mL of acetonitrile.

All samples were finally analyzed by HPLC (Series 1100, Agilent Technologies, Santa Clara, CA, USA) with fluorescence detection (λ_ex/λ_em = 294/404 nm, gain = 12×, injection volume = 1 µL, column temperature = 25 °C). Separation was achieved on a reverse-phase Envirosep PP column (125 × 4.6 mm, 5 µm) using water (A) and acetonitrile (B) at 1 mL min^−1^ (80% B for 15 min, then 100% B for 10 min, maintained for 10 min). After that, 5 min of column re-equilibration was performed by restoring the initial gradient conditions. Analytical blanks and spiked samples (2 μg kg^−1^) were processed identically. Quantification was carried out using calibration curves prepared in acetonitrile with PAH-Mix 9 standard (Dr. Ehrenstorfer GmbH, Augsburg, Germany) at concentrations of 1, 2, 5, 10, 20, 50, 100, and 200 ng mL^−1^. Recoveries for spiked samples ranged from 81% to 90%. The LOQ for all analytes was 0.8 μg kg^−1^. All values were expressed on a w.w. basis.

##### Detection of Non-Dioxin-like Polychlorinated Biphenyls (NDL-PCBs)

The detection of NDL-PCBs (28, 32, 52, 101, 138, 153, 180) was performed following the method previously described [26]. Sample extraction was performed as described in “Detection of Polycyclic Aromatic Hydrocarbons (PAHs)”. Prior to NDL-PCB analysis, a solvent exchange step was performed by evaporating acetonitrile and reconstituting with iso-octane. The detection was performed by gas chromatography–mass spectrometry (GC–MS) using a DSQ 70 FOCUS single quadrupole system (Thermo Fisher Scientific, Waltham, MA, USA) equipped with an AS 3000 autosampler. Helium (>99.9%) was used as carrier gas at 1.2 mL min^−1^. The injector was maintained at 250 °C in splitless mode (1 μL). The oven temperature program started at 100 °C (1 min), increased to 190 °C at 20 °C min^−1^ (2 min hold), then to 250 °C at 3 °C min^−1^ and to 300 °C at 50 °C min^−1^ (20 min hold). The mass spectrometer operated in electron ionization (70 eV) with the transfer line at 270 °C. Data were acquired using Xcalibur software (version 4.8). Quantification was based on peak areas and calibration curves prepared with PCB-Mix, PCB 198, and PCB 155 at concentrations of 5, 10, 50, 100, and 200 ng mL^−1^ in iso-octane. Analytical blanks and two spiked samples (12 μg kg^−1^) were included in each batch to assess method performance. Recoveries ranged from 94% to 101%. The LOQ for all analytes was 1.2 ng g^−1^, and results were expressed on a w.w. basis.

##### Detection of Pesticides

A total of 95 pesticides were investigated (the complete list is available in Appendix A). The analysis was performed following the Swedish ethyl acetate method (SweEt) [27]. Extracts were analyzed by GC–MS/MS using a Thermo Scientific TRACE 1300 gas chromatograph (Thermo Fisher Scientific, Waltham, MA, USA) coupled to a Thermo Scientific TSQ 8000 Evo triple quadrupole mass spectrometer (Thermo Fisher Scientific, Waltham, MA, USA) equipped with an Thermo Scientific AS 3000 Autosampler (Thermo Fisher Scientific, Waltham, MA, USA). Data acquisition and processing were performed using Xcalibur (version 4.8) and TraceFinder (version 5.2), respectively. Separation was achieved on a DB-5MS GC Column (30 m × 0.25 mm, 0.25 μm) using helium as carrier gas (1.2 mL min^−1^). The injector temperature was set at 250 °C in splitless mode with a 1 μL injection volume, and electron ionization was performed at 70 eV. The oven temperature program ranged from 50 °C to 325 °C with a final hold of 2 min. Detection was carried out in Selected Reaction Monitoring (SRM) mode using two transitions per pesticide (quantifier and qualifier). Matrix-matched calibration curves (0.5–10 ppm) were prepared using pesticide-free fish muscle. Quality checks were evaluated in accordance with the SANTE/11312/2021 guidance document [28]. LOQ ranged from 0.002 to 0.005 μg kg^−1^. All results were expressed on a w.w. basis.

##### Microplastics (MPs) Analysis and Assurance/Quality Control

Total intestinal tracts of *S. glanis* and *P. clarkii* were subjected to alkaline digestion. Digestion of the samples was performed using a solution of KOH and NaClO, followed by treatment with H_2_O_2_ after a few minutes to degrade the organic matrix while preserving plastic polymers. The samples were stirred for 12 h to ensure complete digestion. After digestion, the final solutions were filtered through sterile mixed cellulose membrane filters (47 mm diameter, 0.45 μm pore size; Artiglass, Padova, Italy) under an HEPA-It iltered laminar flow hood to prevent airborne contamination. Filters were placed in glass Petri dishes and dried overnight.

Dried filters were first examined using stereomicroscopy (10–80× magnification; SMZ-800 N, Nikon, Tokyo, Japan) to isolate suspected MPs. Chemical identification was then performed by micro-Fourier transform infrared spectroscopy (μFT-IR; Nicolet iN10 MX, Thermo Fisher Scientific, Waltham, MA, USA) operating in reflection mode with an MCT-A detector (spectral range 7800–650 cm^−1^) cooled with liquid nitrogen. Polymer identification was achieved by comparison of spectra with reference libraries of both pristine and weathered MPs (OMNIC^TM^ Picta^TM^ libraries and in-house spectral databases), applying a match threshold greater than 80%. The detection limit corresponded to an MP size of 10 μm. Identified MPs were categorized according to color, polymer type, and size following established classification criteria [29].

Calculations were performed for each species and site to assess the risks of MPs using the Polymer Hazard Index (PHI) and the following formula:

(4) PHI=ΣPn×Sn

where Pn is the relative proportion of each polymer in the analyzed samples and Sn represents the hazard score assigned to each polymer type [30]. Based on the methodology of Ranjani et al. [31], the calculated PHI values were used to assign each sample of plastic debris to its corresponding hazard category and risk level. Additional details on PHI calculation are detailed in Supplementary Material (Text S2).

The quality assurance and quality control procedures applied in this study followed the main recommendations reported in the literature [32]. The Bioscience Research Centre is an ACCREDIA-accredited laboratory (accreditation n. 01403) authorized to perform MPs analyses (test method IO 004.31) in different matrices, including biological samples. The laboratory operates under controlled conditions, with appropriate equipment and standardized procedures designed to minimize sample contamination and ensure the reliability and quality of the analytical process. Sample preparation and processing were performed in a clean environment under an HEPA-filtered laminar flow hood and inside a glove box (SGS20-13599 Glove Box, Iteco Engineering, Ravenna, Italy) to minimize environmental contamination. Materials and equipment were cleaned after each analysis, and water and reagents were pre-filtered before use. All water and reagents used for sample rinsing and extraction were pre-filtered through 0.45 μm cellulose acetate membranes filters (47 mm diameter, 0.45 μm pore size; Artiglass, Padova, Italy) before use.

Airborne contamination was monitored using blank fiber filter disks (N = 5) placed inside the glove box during sample processing. Procedural blanks (N = 5), consisting of all reagents used during sample preparation and analysis but without the biological matrix, were processed through the entire analytical workflow and included as negative controls to assess potential contamination arising from reagents, laboratory handling, and analytical procedures. No MPs were detected in either the airborne contamination controls or the procedural blanks, confirming the absence of background contamination during sample preparation and analysis. Consequently, no blank correction was required or applied to the reported MP abundances. A positive procedural control (recovery test) was performed in the laboratory to control the quality of the analysis. MP extraction showed a recovery rate of 87.0%.

Instrument performance of the μFT-IR system (Thermo Scientific Nicolet iN10 MX, Thermo Fisher Scientific, Waltham, MA, USA) was subjected to strict quality control procedures, including biannual calibration using polystyrene reference standards (Thermo^®^, Instrument Qualification Kit, Thermo Fisher Scientific, Waltham, MA, USA) and daily per-formance verification using certified polymer MPs (Polypropylene-PP, Polyethylene-PE, Polyvinyl Chloride-PVC, and Polyethylene Terephthalate-PET) prior to each analytical session to confirm instrument precision and spectral accuracy.

### 2.5. Statistical Analysis

Data were analyzed using R software (v.4.2.2) and RStudio (2022.07.2 + 576). Normality (Shapiro–Wilk) and homogeneity of variances (Levene’s test) were assessed, and data were found not to meet parametric assumptions; therefore, non-parametric tests were applied.

#### 2.5.1. Univariate Analysis

Trace elements and REEs were expressed as mg/kg (log10) and visualized with boxplots (ggplot2 package). Differences in contamination of *S. glanis* between Great Lake and Small Lake were evaluated using Kruskal–Wallis tests with Bonferroni adjustment (*p* < 0.05—agricolae package). In addition, descriptive statistics, including mean ± standard deviation (SD), median, interquartile range (IQR), minimum–maximum range, and effect size, were calculated for each trace element and REE. For trace elements and REEs, values between the LOD and LOQ were included in the statistical analyses. Values below the LOD were considered equal to LOD. Spearman’s rank correlation coefficient (rho) between total weight and trace element/REE concentrations were calculated for both *S. glanis* and *P. clarkii* (stats package) and visualized using heatmaps and scatterplots (for elements showing significant correlations). Organic contaminants were analyzed separately from other contaminant classes. Due to the very low detection frequency, only compounds with concentrations above the LOQ were considered for data reporting and statistical treatment. For this contaminant class, only descriptive statistics (mean values) were calculated, while no inferential statistical analyses were performed. MP concentrations were expressed per intestinal tract, and lake differences in *S. glanis* were assessed with Kruskal–Wallis tests with Bonferroni adjustment (*p* < 0.05—agricolae package). For both species, the frequency (%) of MPs by color and polymer type, as well as the PHI, were visualized using bar plots, while correlations between total weight and MPs concentrations were assessed using Spearman’s rank correlation coefficient (rho) (stats package) and shown with scatterplots. No correction for multiple testing was applied in the Spearman’s rank correlation analysis, as the analyses were exploratory.

#### 2.5.2. Multivariate Analysis

Data were extracted from the dataset and analyzed using non-metric multidimensional scaling (NMDS) to explore multivariate differences among samples according to species (*S. glanis* and *P. clarkii*) and sampling sites (Great Lake and Small Lake). The chemical matrix included trace elements, REEs, and MP concentrations. NMDS was performed on the standardized chemical matrix using the metaMDS function in the vegan package, with Euclidean distance, two ordination dimensions (k = 2), and a maximum of 200 random starts (trymax = 200). Automatic data transformation within metaMDS was disabled because transformations had already been applied manually. Ordination quality was assessed using the NMDS stress value. Ordination results were visualized using ggplot2, with samples plotted according to NMDS axis scores.

Multivariate differences in elemental profiles were evaluated using Permutational Multivariate Analysis of Variance (PERMANOVA) implemented in the adonis2 function of the vegan package in R. Two separate analyses were performed to avoid potential confounding effects associated with the unbalanced distribution of species among sampling sites.

The first analysis assessed site-related differences using only *S. glanis* specimens collected from Great Lake and Small Lake. The second analysis evaluated species-related differences within the same environmental context by comparing *S. glanis* and *P. clarkii* specimens collected exclusively from Great Lake.

Prior to analysis, data were converted to numeric values, variables containing exclusively missing values or zero variance were removed, and missing values were imputed using the median value of each variable. Data were subsequently log-transformed using the log1p function and standardized by z-score scaling. Euclidean distance matrices were then calculated from the transformed datasets.

Statistical significance was assessed using 999 permutations. To verify the assumption of homogeneity of multivariate dispersion among groups, a PERMDISP analysis followed by ANOVA was additionally performed for each comparison.

## 3. Results

### 3.1. Trace Elements and REEs

**Figure 2:**
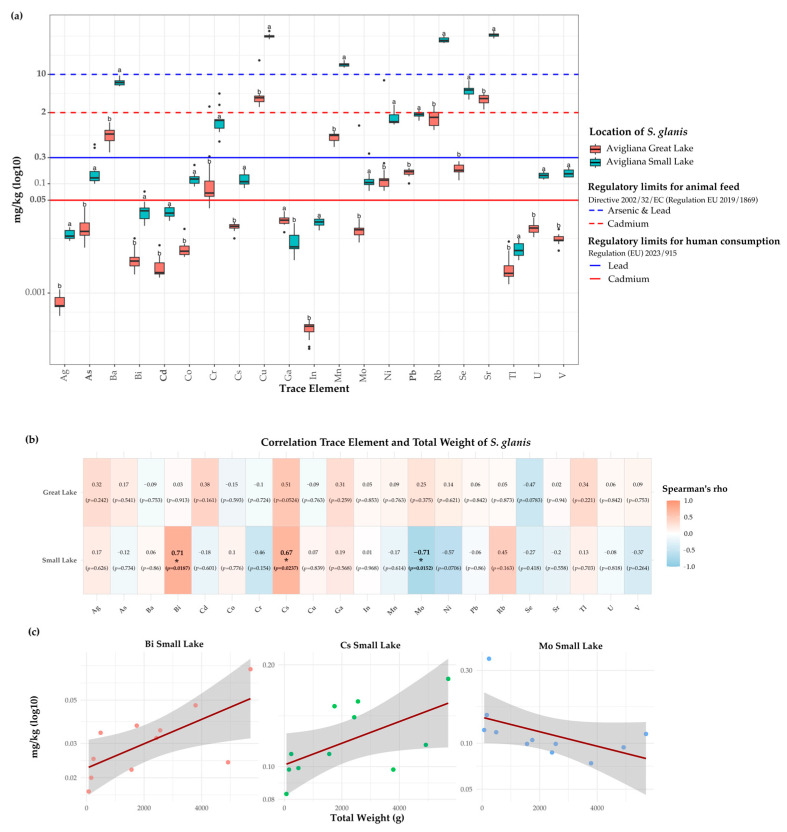
(**a**) Trace element concentrations (mg/kg w.w., log10) in *S. glanis* muscle from Avigliana Great Lake (orange) and Small Lake (light blue). Letters indicate significant differences between lakes (Kruskal–Wallis test with Bonferroni correction). Dashed lines: regulatory limits for animal feed (Directive 2002/32/EC; blue = As and Pb, red = Cd). Solid lines: regulatory limits for human consumption in fish muscle (Regulation EU 2023/915; blue = Pb, red = Cd). (**b**) Heatmap showing the Spearman’s correlation matrix between trace element concentrations and total weight of *S. glanis* from the Great Lake and the Small Lake (* *p* ≤ 0.05). (**c**) Scatterplots showing the relationships between total weight and the concentrations of trace elements (Bi, Cs, and Mo) that were significantly correlated in *S. glanis* from the Small Lake.

In *S. glanis*, 20 out of 21 analyzed elements exhibited significantly higher concentrations in Small Lake compared to Great Lake, with the sole exception of Ga (Figure 2a). The results of the Kruskal–Wallis tests with Bonferroni correction comparing trace element concentrations in *S. glanis* from Avigliana Great Lake and Avigliana Small Lake are reported in Table S1 (Supplementary Material), together with descriptive statistics (mean ± SD, median, interquartile range, minimum–maximum range) and effect size values. In Great Lake, the least abundant element was In (0.00030 ± 0.000005 mg/kg), while Cu was the most abundant (4.5 ± 3.78 mg/kg). In Small Lake, Tl was the least frequently detected element (0.0066 ± 0.00207 mg/kg), whereas Sr was the most abundant (52.9 ± 5.06 mg/kg). Considering metals regulated in fish muscle for animal feed (As, Cd, Pb) according to Directive 2002/32/EC [33], amended by Regulation EU 2019/1869 [34], and for human consumption (Pb and Cd) Regulation EU 2023/915 [35], the average concentrations measured in *S. glanis* muscle followed the order: Pb (Great Lake: 0.16 ± 0.024 mg/kg; Small Lake: 1.86 ± 0.266 mg/kg) > As (Great Lake: 0.017 ± 0.0096 mg/kg; Small Lake: 0.19 ± 0.148 mg/kg) > Cd (Great Lake: 0.003 ± 0.0015 mg/kg; Small Lake: 0.030 ± 0.0067 mg/kg).

Spearman’s correlation patterns differed markedly between the two study sites (Figure 2b). In the Great Lake, no trace element showed a significant association with the total weight of *S. glanis* individuals. In contrast, the Small Lake population displayed clear weight-dependent trends for three elements: Bi and Cs were positively correlated with total weight, indicating higher concentrations in larger individuals, whereas Mo showed a pronounced negative correlation, with decreasing concentrations as body weight increased. These relationships are further illustrated in Figure 2c, where the scatterplots confirm the upward trends for Bi and Cs and the inverse pattern for Mo.

**Figure 3:**
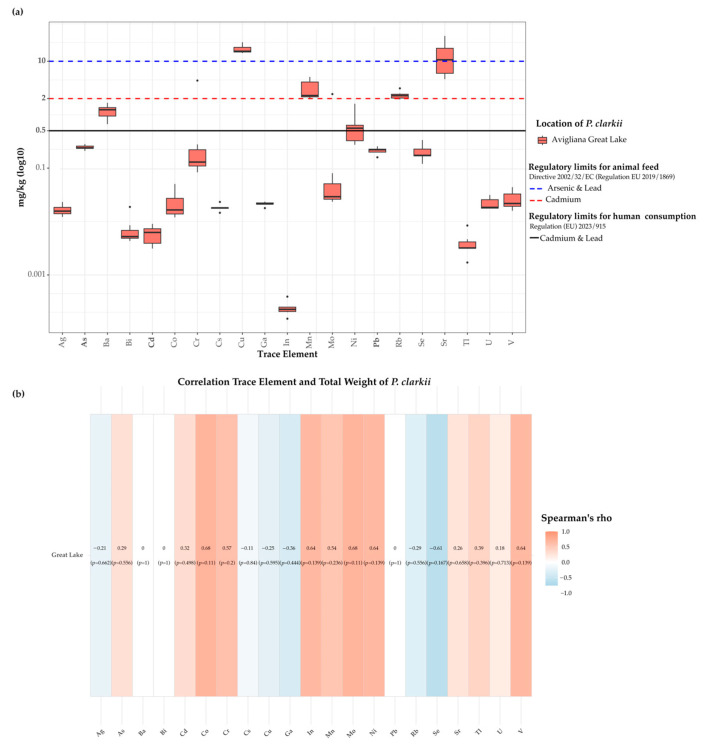
(**a**) Trace element concentrations (mg/kg w.w., log10) in *P. clarkii* muscle from Avigliana Great Lake (orange). Dashed lines: regulatory limits for animal feed (Directive 2002/32/EC; blue = As and Pb, red = Cd). Solid lines: regulatory limits for human consumption in crustacean matrix (Regulation EU 2023/915; black = Cd and Pb). (**b**) Heatmap showing the Spearman’s correlation matrix between trace element concentrations and total weight of *P. clarkii* from the Great Lake.

Figure 3a shows the concentrations of trace elements in *P. clarkii* specimens collected from Great Lake. The least represented element was In (0.0003 ± 0.00004 mg/kg), whereas the most abundant element was Cu, with an average concentration of 16.9 ± 3.15 mg/kg. Descriptive statistics (mean ± SD, median, interquartile range, minimum–maximum range) are reported in Table S2 (Supplementary Material). The concentration ranking of metals regulated for animal feed (As, Cd, Pb) and human consumption (Cd and Pb), considering the crustacean matrix, was: As (0.25 ± 0.023 mg/kg) > Pb (0.21 ± 0.030 mg/kg) > Cd (0.006 ± 0.0023 mg/kg) [33,35].

The correlation analysis performed on *P. clarkii* from the Great Lake did not reveal any significant associations between trace element concentrations and total weight (Figure 3b). Spearman’s rho values were generally low and inconsistent across elements, and none of the correlations reached statistical significance. This indicates that, within this population, no statistically significant association between body weight and trace element accumulation was detected.

**Figure 4:**
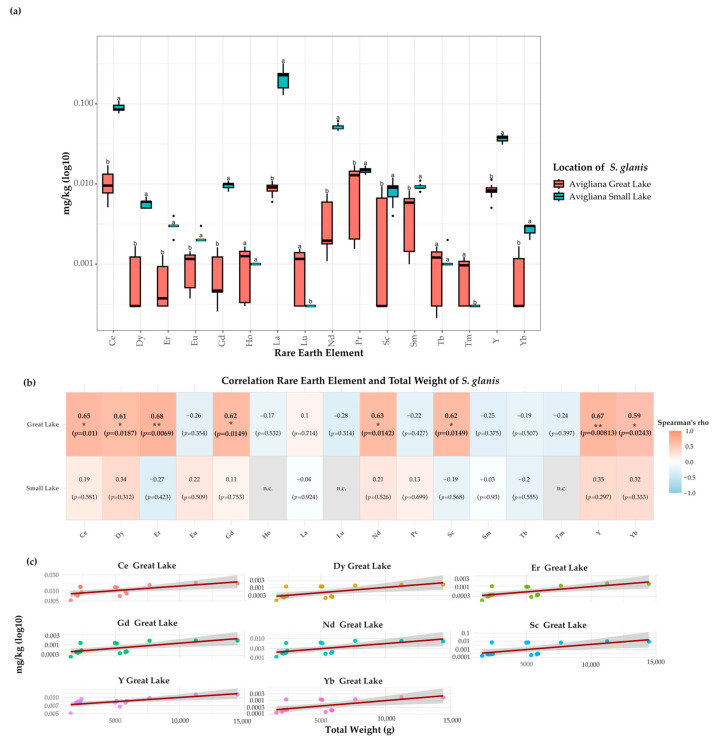
(**a**) Rare earth element concentrations (mg/kg w.w., log10) in *S. glanis* muscle from Avigliana Great Lake (orange) and Small Lake (light blue). Letters indicate significant differences between lakes (Kruskal–Wallis test with Bonferroni correction). (**b**) Heatmap showing the Spearman’s correlation matrix between rare earth element concentrations and total weight of *S. glanis* from the Great Lake and the Small Lake, Spearman (* *p* ≤ 0.05, ** *p* ≤ 0.01; ‘n.c.’ indicates not computable correlations due to zero variance). (**c**) Scatterplots showing the relationships between total weight and the concentrations of rare earth elements (Ce, Dy, Er, Gd, Nd, Sc, Y, and Yb) that were significantly correlated in *S. glanis* from the Great Lake.

REEs analyses in *S. glanis* (Figure 4a) revealed overall higher concentrations in samples from Small Lake compared to those from Great Lake (ΣREEs: 0.445 mg/kg vs. 0.056 mg/kg). For 12 out of 16 elements, significant higher concentrations were observed in Small Lake, whereas Ho and Tb showed similar values between the two lakes. In contrast, Lu and Tm were found at higher levels in Great Lake. The results of the Kruskal–Wallis tests with Bonferroni correction comparing REE concentrations in *S. glanis* from Avigliana Great Lake and Avigliana Small Lake are reported in Table S3 (Supplementary Material), together with descriptive statistics (mean ± SD, median, interquartile range, minimum–maximum range) and effect size values. In samples from Great Lake, Ce was the most abundant element (0.011 ± 0.0038 mg/kg), while Er was the least abundant (0.0006 ± 0.0004 mg/kg). In samples from Small Lake, La exhibited the highest concentration (0.208 ± 0.0611 mg/kg), whereas Lu and Tm showed the lowest concentrations (0.0003 ± 0 mg/kg). The mean ratio of light-to-heavy rare earth elements (LREEs:HREEs; LREEs = La, Ce, Pr, Nd, Sm; HREEs = Eu, Gd, Tb, Dy, Ho, Er, Tm, Yb, Lu, Y) in *S. glanis* was 2.35 (N = 15, range 1.90–2.91) in specimens from Great Lake and 5.86 (N = 15, range 1.90–7.10) in those from Small Lake. The correlation analysis revealed site-specific patterns in the relationship between REE concentrations and total body weight of *S. glanis* (Figure 4b). In the Great Lake population, eight out of sixteen REEs (Ce, Dy, Er, Gd, Nd, Sc, Y, and Yb) exhibited significant positive correlations with body weight, indicating that heavier individuals accumulated higher concentrations of these elements. In contrast, no significant correlations were observed in the Small Lake population. The scatterplots in Figure 4c further illustrate the weight-dependent trends for selected REEs in the Great Lake.

**Figure 5:**
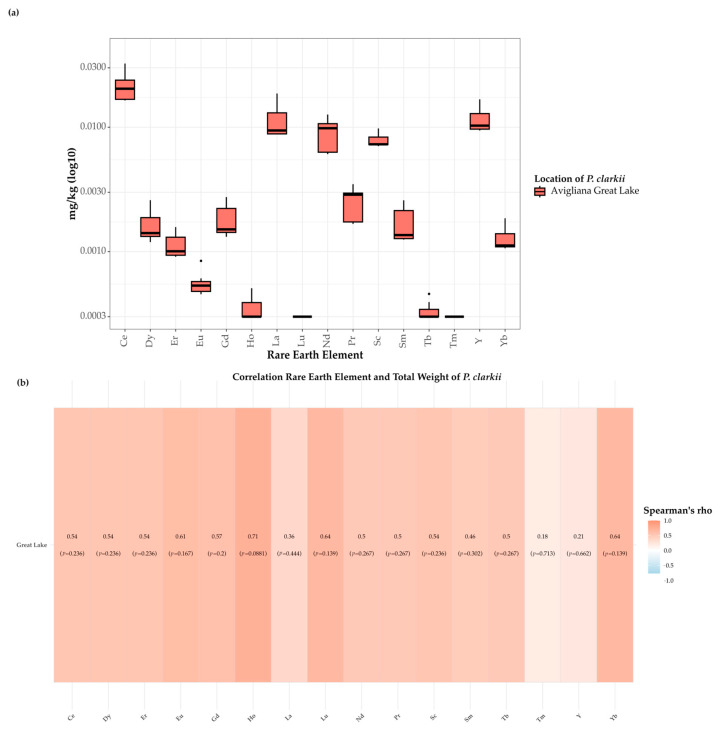
(**a**) Rare earth element concentrations (mg/kg w.w., log10) in *P. clarkii* muscle from Avigliana Great Lake (orange). (**b**) Heatmap showing the Spearman’s correlation matrix between rare earth element concentrations and total weight of *P. clarkii* from the Great Lake.

In *P. clarkii* from Great Lake, the total concentration of REEs was 0.074 mg/kg (Figure 5a). The most abundant element was Ce (0.02 ± 0.006 mg/kg), while the least abundant were Lu and Tm (0.00030 ± 0 mg/kg). LREEs:HREEs was 2.36 (N = 40, range 1.95–3.14). Descriptive statistics (mean ± SD, median, interquartile range, minimum–maximum range) are reported in Table S4 (Supplementary Material).

Chondrite-normalized REE patterns revealed clear species- and site-specific differences (Figure S1—Supplementary Material).

*S. glanis* displayed marked differences between sites. In Great Lake, normalized REE concentrations were lower and characterized by irregular, non-monotonic patterns with high variability among samples. A pronounced positive Eu anomaly (Eu/Eu* ≈ 1.78) and a strong negative Ce anomaly (Ce/Ce* ≈ 0.47) were observed. In Small Lake, *S. glanis* showed higher normalized REE concentrations and a more structured pattern, with a gradual decrease from LREEs (La-Nd) to HREEs (Dy-Lu). Despite the more coherent profile, negative Ce and Eu anomalies persisted (Ce/Ce* ≈ 0.40; Eu/Eu* ≈ 0.70).

In contrast, for *P. clarkii* from Great Lake, REE profiles showed a smooth and consistent decrease from LREEs to HREEs. The normalized patterns were regular and closely aligned among samples, indicating low variability. Ce and Eu anomalies were close to unity (Ce/Ce* ≈ 0.97; Eu/Eu* ≈ 0.98), suggesting the absence of significant fractionation and reflecting environmental REE distributions.

### 3.2. Organic Contaminants: PAHs, NDL-PCBs, and Pesticides

PAHs in *S. glanis* were below the LOQ for all compounds, except for chrysene, which was detected in a single sample at 0.9 µg/kg. In *P. clarkii*, PAHs were also below the LOQ, with the exception of chrysene, which showed a mean concentration of 0.9 µg/kg (range: <0.8–1.3 µg/kg, only one sample was below LOQ, and was treated equal to LOQ). All NDL-PCBs and pesticide compounds were below the LOQ in both species.

### 3.3. Microplastics (MPs)

**Figure 6:**
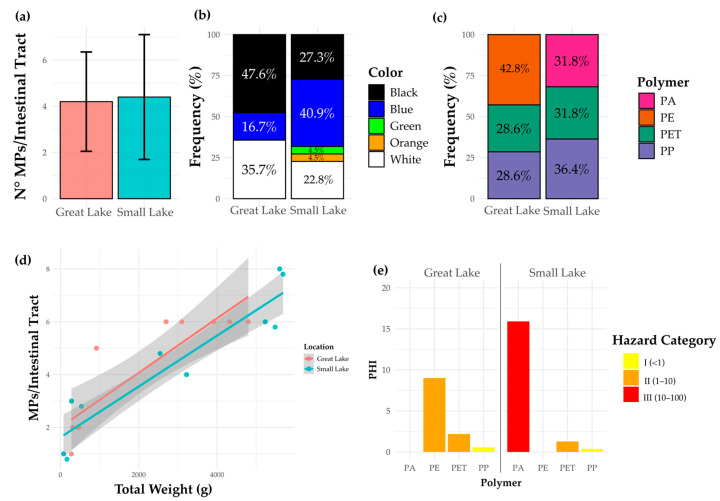
Microplastics in *S. glanis* from Avigliana Great Lake and Avigliana Small Lake: (**a**) Concentration of MPs in intestinal tract expressed as number of MPs/intestinal tract w.w.; (Kruskal–Wallis test with Bonferroni correction, *p* < 0.05). (**b**) Frequency (%) of color distribution (Great Lake: 47.6% = 2 MPs/intestinal tract; 35.7% = 1.5 MPs/intestinal tract; 16.7% = 0.7 MPs/intestinal tract; Small Lake: 40.9% = 1.8 MPs/intestinal tract; 27.3% = 1.2 MPs/intestinal tract; 22.8% = 1 MPs/intestinal tract; 4.5% = 0.2 MPs/intestinal tract). (**c**) Frequency (%) of polymer distribution (Great Lake: 42.8% = 1.8; 28.6% = 1.2 MPs/intestinal tract; Small Lake: 36.4% = 1.5 MPs/intestinal tract; 31.8% = 1.3 MPs/intestinal tract), (PA = polyamide; PE = polyethylene; PET = polyethylene terephthalate; PP = polypropylene). (**d**) Relationship between total weight (g) of *S. glanis* and number of MPs/intestinal tract. (**e**) Polymer Hazard Index (PHI) of polymers detected in *S. glanis* from Great Lake and the Small Lake (PA = polyamide; PE = polyethylene; PET = polyethylene terephthalate; PP = polypropylene).

No significant differences in MP concentrations in the intestinal tracts of *S. glanis* were observed between Avigliana Great Lake and Avigliana Small Lake (Great Lake: 4.2 ± 2.15 MPs/intestinal tract; Small Lake: 4.4 ± 2.70 MPs/intestinal tract; *p* > 0.05; Figure 6a). Results of the Kruskal–Wallis tests with Bonferroni correction comparing MPs/intestinal tract concentrations in *S. glanis* from Avigliana Great Lake and Avigliana Small Lake are reported in Table S5 (Supplementary Material), together with descriptive statistics (mean ± SD, median, interquartile range, minimum–maximum range) and effect size values.

Regarding color distribution, black, white, and blue MPs were the most prevalent in both species across the two sampling sites. Low levels of orange and green MPs were detected only in *S. glanis* from Avigliana Small Lake (Figure 6b).

In terms of polymer composition, MPs in Great Lake were PE, followed by PET and PP. In contrast, in Small Lake, PP was the most abundant polymer, followed by PET and polyamide (PA) (Figure 6c).

Figure 6d shows a positive relationship between fish weight and MP concentrations in both lakes. This relationship was statistically significant for Avigliana Great Lake (Spearman’s rho = 0.927, *p* < 0.001, N = 15) and Avigliana Small Lake (Spearman’s rho = 0.946, *p* < 0.001, N = 15), indicating that larger individuals tend to contain more MPs.

Figure 6e shows the distribution of PHI for MPs detected in *S. glanis* from both Great and Small Lakes. In Great Lake PP (mean 1.2 MPs/intestinal tract) was classified as Category I (<1), indicating low-hazard polymers, and PET (mean 1.2 MPs/intestinal tract) and PE (mean 1.8 MPs/intestinal tract) as Category II (1–10), reflecting moderate hazard. In Small Lake, PA (mean 1.3 MPs/intestinal tract) exhibited the highest hazard, falling into Category III (10–100), while PET (mean 1.3 MPs/intestinal tract) and PP (mean 1.5 MPs/intestinal tract) remained in Categories II and I, respectively, showing a hazard pattern similar to that observed in the Great Lake for these polymers.

Finally, regarding size, the mean MP lengths in *S. glanis* were 252 ± 100.5 µm in Great Lake (range: 108–442 µm) and 250 ± 64.1 µm in Small Lake (range: 153–361 µm).

**Figure 7:**
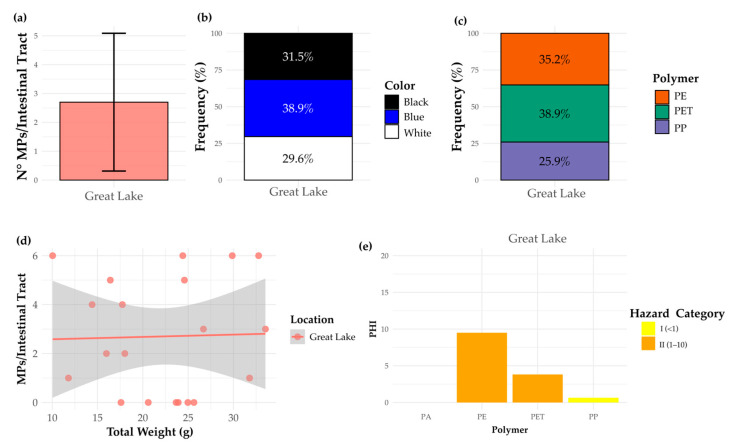
MPs in *P. clarkii* from Avigliana Great Lake. (**a**) Concentration of MPs in intestinal tract expressed as number of MPs/ intestinal tract w.w. (**b**) Frequency (%) of color distribution (38.9% = 1.1 MPs/intestinal tract; 31.5% = 0.9 MPs/intestinal tract; 29.6% = 0.8 MPs/intestinal tract). (**c**) Frequency (%) of polymer distribution (38.9% = 1.1 MPs/intestinal tract; 35.2% = 0.9 MPs/intestinal tract; 25.9% = 0.7 MPs/intestinal tract) (PE = polyethylene; PET = polyethylene terephthalate; PP = polypropylene). (**d**) Relationship between total weight (g) of *P. clarkii* and number of MPs/intestinal tract. (**e**) Polymer Hazard Index (PHI) of polymers detected in *P.clarkii* from Great Lake (PA = polyamide; PE = polyethylene; PET = polyethylene terephthalate; PP = polypropylene).

In *P. clarkii* collected from Great Lake, the mean MP concentration was 2.7 ± 2.39 MPs per intestinal tract (Figure 7a). Descriptive statistics (mean ± SD, median, interquartile range, minimum–maximum range) are reported in Table S6. Blue MPs were the most abundant (38.9%), followed by black (31.5%) and white (29.6%) (Figure 7b). Regarding polymer composition, PET was dominant (38.9%), followed by PE (35.2%) and PP (25.9%) (Figure 7c). No significant correlation was found between body weight and MP concentrations (Spearman’s rho = 0.0322, *p* = 0.893, N = 40).

Figure 7e shows the PHI of MPs detected in *P. clarkii* from Great Lake. PE (mean 0.9 MPs/intestinal tract) and PET (mean 1.1 MPs/intestinal tract) fell within Category II (1–10), indicating polymers of moderate hazard, whereas PP (mean 0.7 MPs/intestinal tract) was classified in Category I (<1).

The mean MP length was 294 ± 110.6 µm, ranging from 100 to 446 µm.

### 3.4. Multivariate Analysis

**Figure 8:**
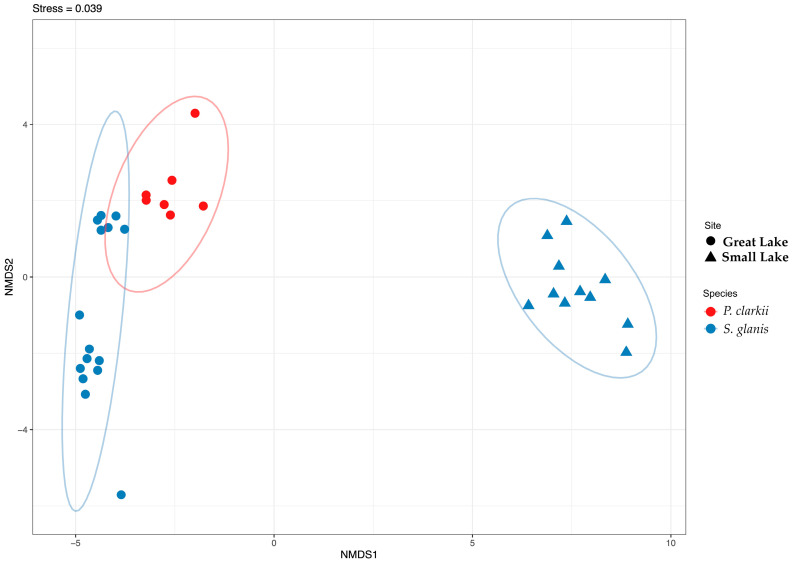
Non-metric multidimensional scaling (NMDS) based on a chemical matrix including trace elements, rare earth elements (REEs), and microplastic (MP) concentrations. Samples are grouped by sampling site (circles = Great Lake; triangles = Small Lake) and species (*S. glanis* = blue, *P. clarkii* = red). Ellipses indicate group dispersion.

The NMDS ordination based on chemical variables showed a clear separation among samples (Figure 8), with a very low stress value (stress = 0.039), indicating an excellent representation of the multivariate structure in two dimensions. A strong spatial pattern was observed along the first NMDS axis (NMDS1), which clearly separated samples from the two sites. Specimens collected in Small Lake clustered distinctly on the right side of the ordination space, forming a well-defined group with limited dispersion. In contrast, samples from Great Lake were located on the left side of the plot, showing a broader spread along the second axis (NMDS2), indicating higher within-site variability in chemical composition. Within Great Lake, a partial separation between species was evident. *P. clarkii* formed a relatively cohesive cluster in the upper portion of the ordination space, whereas *S. glanis* showed a wider dispersion, particularly along NMDS2, suggesting greater variability in chemical profiles for this species at this site. The ellipses further highlighted this pattern, with *S. glanis* exhibiting a larger spread compared to *P. clarkii*.

PERMANOVA revealed significant site-related differences in elemental profiles of *S. glanis* between the two lakes (Pseudo-F = 65.56, R^2^ = 0.732, *p* = 0.001), with the sampling site explaining 73.2% of the total multivariate variance. The PERMDISP analysis showed no significant differences in dispersion between sites (F = 1.29, *p* = 0.268), indicating that the PERMANOVA results were primarily driven by differences in centroid location rather than by heterogeneous within-group variability.

Within Great Lake, significant differences in elemental composition were also observed between *S. glanis* and *P. clarkii* (Pseudo-F = 10.44, R^2^ = 0.343, *p* = 0.001), with species identity explaining 34.3% of the total variance. PERMDISP analysis did not detect significant differences in multivariate dispersion between species (F = 0.009, *p* = 0.924), supporting the robustness of the PERMANOVA results.

## 4. Discussion

IAS as *S. glanis* and *P. clarkii* pose a significant threat to native biodiversity and require effective management [36]. At the same time, IAS eradicated can provide valuable insights as bioindicators of environmental contamination and may be considered as alternative feed/food sources [2,16,17]. Assessing the levels of contaminants (inorganic, organic, and emerging) in these species is therefore essential to evaluate the environmental quality and to determine the safety of their potential use as alternative nutritional sources.

### 4.1. Trace Elements and REEs

Trace elements present in the environment originate from numerous anthropogenic activities, including industrial discharges, urban runoff, maritime traffic, and oil extraction operations. Even at very low concentrations, these contaminants can be harmful, causing direct damage to cellular structures, alterations in metabolic processes, and impairment of organism growth and development [37]. Fish and benthonic macroinvertebrates are well-known for their tendency to bioaccumulate trace elements in their muscles [38].

In addition to classical approaches based on chemical analyses of abiotic matrices, increasing attention has been devoted to biological monitoring techniques for assessing trace element contamination in aquatic environments. Active biomonitoring approaches, including the use of standardized biological matrices exposed under controlled conditions, have proven effective in integrating spatial and temporal variability of pollutants and providing ecologically relevant information on bioavailable fractions of pollutants. Recent studies have further highlighted the applicability of such methods for the assessment of heavy metal accumulation in freshwater systems using different biological organisms as exposure tools [39].

Samples of *S. glanis* collected from the two Avigliana Lakes generally showed significantly higher concentrations of trace elements in individuals from Small Lake compared to those from Great Lake (20 out of 21). This difference is consistent with findings reported in the “Dossier Laghi—Lo stato dei Laghi in Piemonte” [40], where Small Lake was classified as heavily polluted or polluted in two out of three sampling sites. In contrast, the two monitored sites in Great Lake were within acceptable limits, indicating overall better environmental conditions. The agreement between environmental quality classifications and bioaccumulation data strengthens the reliability of using IAS as bioindicators.

The observed higher concentrations of trace elements in *S. glanis* from Small Lake may be explained by differences in hydrological and environmental characteristics between the two systems. Despite its more natural surrounding landscape, Small Lake is located at a higher elevation and receives direct inflows from the Trana peat bog and surrounding hillside streams, which likely enhance the input and deposition of organic matter and associated contaminants, promoting its role as a preferential sink for pollutants. In contrast, Great Lake is characterized by a larger water volume and a higher water renewal rate, which may facilitate contaminant dilution and reduce residence time. In addition, lake management interventions such as hypolimnetic water withdrawal for irrigation purposes have contributed to progressive improvements in water quality in Great Lake, a measure not implemented in Small Lake, which may partly explain the observed differences in contaminant accumulation [41,42].

For *P. clarkii*, a comparison between the two lakes was not possible, as this IAS is not yet present in the Small Lake, and therefore currently poses no threat to this lake biodiversity [19].

When comparing trace element concentrations with the limited available literature, the study by Dirican [43] on *S. glanis* from the Kılıçkaya Reservoir (Turkey) provides a useful reference. In specimens from Great Lake, four of the nine analyzed elements (As, Cd, Co, and Se) showed lower concentrations than those reported by Dirican [43], whereas the remaining five (Cr, Cu, Mn, Ni, and Pb) were higher. In contrast, all nine elements (As, Cd, Co, Cr, Cu, Mn, Ni, Pb, and Se) measured in specimens from Small Lake exceeded the concentrations reported in the literature, indicating a markedly higher accumulation of trace elements. This pattern clearly highlights the more severe contamination of Small Lake, plausibly linked to its historical eutrophication, limited hydrodynamic exchange, and enhanced capacity for contaminant accumulation and retention within sediments, with subsequent transfer through the trophic web [42].

Compared to Espinoza et al. [44], who analyzed pooled organs (gills, hepatopancreas, and abdominal muscle) from red swamp crayfish in the Pantanos de Villa wetland (Lima, Peru), the present study showed higher concentrations of the common trace elements analyzed (Cu, Ni and Pb). In comparison with Li et al. [45], who reported mean concentrations in muscle from 4709 *P. clarkii* across 11 Chinese provinces (2010–2020), As, Cd, Cr, and Se were lower in the present study, while Pb was higher reflecting local environmental conditions.

Spearman’s correlations differed between the two sites. In *S. glanis*, no significant associations with body weight were found in the Great Lake population, while in the Small Lake population, Bi and Cs increased with size and Mo decreased. In *P. clarkii* from the Great Lake, trace element accumulation was independent of body weight. These patterns are consistent with Balzani et al. [46], who reported size-dependent accumulation in predatory fish (*S. glanis* for Fe and Co) but weak or absent correlations in *P. clarkii*. Overall, these results indicate that size partially influences metal accumulation in predatory species, whereas in benthic omnivores like *P. clarkii*, accumulation is no size-dependent.

Regarding the potential use of *S. glanis* and *P. clarkii* as alternative animal feed/food, trace elements regulated under Directive 2002/32/EC (as amended by Regulation EU 2019/1869) [33,34] include As, Cd, and Pb, with maximum permitted levels of 10 mg/kg for As and Pb, and 2 mg/kg for Cd. All analyzed samples of *S. glanis* from both lakes, as well as *P. clarkii* samples from Great Lake, showed concentrations below these regulatory limits (Figure 2a and Figure 3a). The measured values were, in most cases, below the limits established for human consumption under Regulation (EU) 2023/915 [35], which sets maximum levels for Cd at 0.05 mg/kg in fish muscle and 0.5 mg/kg in crustaceans, and for Pb at 0.3 mg/kg in fish muscle and 0.5 mg/kg in crustaceans. The only exception was *S. glanis* from Small Lake, in which Pb concentration (1.86 ± 0.266 mg/kg) exceeded the limit established for fish muscle (0.3 mg/kg). The regulation (EU) 2023/915 [35] was recently updated by a Regulation (EU) 2025/1891 [47], which introduced maximum limits for inorganic As in fish muscle and crustaceans (0.10 mg/kg). However, since only total As was measured, a direct comparison with the maximum levels for inorganic As is not possible without performing As speciation analysis.

Passing to REEs, they are primarily released into the environment as a result of anthropogenic activities, including electronics, renewable energy production, and manufacturing industries. Although generally found at low concentrations, their accumulation in organisms may lead to adverse effects such as oxidative stress, ionic imbalance, enzyme inhibition, and reproductive toxicity [48].

Similar to trace elements, concentrations of REEs in *S. glanis* were generally higher in individuals from Small Lake compared to those from Great Lake, further confirming the greater contamination of Small Lake [40,42]. These results support the role of this IAS as potential bioindicator of environmental contaminants.

The ΣREEs in *S. glanis* from Great Lake and Small Lake of Avigliana (0.056 mg/kg and 0.445 mg/kg, respectively) were markedly higher than those measured in muscle tissues of *S. glanis* from the Po River (0.005 mg/kg at Moncalieri and 0.007 mg/kg at Murazzi), as reported by Pastorino et al. [49], who investigated REE concentrations in fish communities across three sampling sites (Moncalieri, Murazzi, and San Mauro), considering both native and non-native species. However, in agreement with that study, the most abundant REEs in both lakes were La, Ce, Nd, and Y. The LREEs:HREEs ratio was higher in the present study, with mean values of 2.35 (range: 1.90–2.91) in specimens from Great Lake and 5.86 (range: 1.90–7.10) in those from Small Lake. In comparison, Pastorino et al. [49] reported a lower mean L:H ratio of 1.25 (range: 0.74–3.56).

To the best of our knowledge, this is the first study investigating *P. clarkii* as a bioindicator for the accumulation of REEs. Currently, a limited number of studies have examined REE bioaccumulation in this species restricted to controlled exposure experiments in which specific REEs, as Gd and La, were administered to *P. clarkii* at known concentrations [50,51]. The study most closely related to our work investigated macroinvertebrate communities sampled from six sites in Northeast Italy, which were predominantly composed of insects (Hexapoda, 76.05–99.66%), except for site five, which was dominated by Malacostraca (64.71%). In that study, ΣREEs ranged from 1.95 to 7.05 mg/kg, whereas in *P. clarkii* from Great Lake of Avigliana we observed much lower levels (0.074 mg/kg) [52]. This marked difference likely reflects differences in trophic habits, sediment ingestion, and exposure pathways. In agreement with that work, the most abundant REEs were Ce, La, Y, and Nd.

Spearman’s correlation analysis revealed a stronger relationship between body weight and REE concentrations in *S. glanis* from the Great Lake of Avigliana, with significant correlations observed for eight out of sixteen REEs. In contrast, only three out of twenty-one trace elements were significantly correlated with weight in the same species, with Mo showing a negative correlation. These results indicate a stronger association between body weight and REE accumulation compared to trace elements in *S. glanis*. Conversely, no significant correlations between body weight and REE concentrations were observed in *S. glanis* from the Small Lake or in *P. clarkii* from the Great Lake, a pattern that was also observed for trace elements in *P. clarkii* at the same sampling site. To date, there are no published studies investigating the relationship between body weight and REE accumulation in these species, highlighting the novelty of our findings.

No regulatory thresholds are currently established for fish or crustaceans regarding REEs in feed and food sources reflecting the current lack of regulatory frameworks for these emerging pollutants, which limits the ability to fully assess their potential risks.

Chondrite-normalized REE patterns revealed a dual control on REE distribution, driven by both environmental availability and species-specific biological processes [53,54].

*S. glanis* exhibited pronounced deviations from these patterns, particularly in Great Lake. The irregular REE profiles and strong anomalies indicate that biological regulation plays a significant role in REE accumulation, as REE bioaccumulation and fractionation are known to be strongly species-specific and influenced by feeding behavior and physiology [55], with evidence that REE patterns can either reflect environmental signatures or show organism-driven deviations and anomalies depending on species and tissue characteristics [49,56].

Negative Ce anomalies are typically associated with oxic conditions, where Ce(III) is oxidized to Ce(IV) and preferentially removed from solution, while Eu anomalies may reflect redox conditions or source-related effects [57]. However, the higher variability observed in *S. glanis* suggests that physiological processes, such as selective uptake and tissue-specific binding, may dominate over environmental signals, especially at low exposure levels, as REE accumulation is strongly influenced by species–specific biological traits and internal distribution processes [55,58].

In Small Lake, *S. glanis* showed higher REE concentrations and more regular patterns, suggesting a stronger influence of environmental conditions. The shift from irregular to more consistent REE profiles with increasing concentrations indicates that environmental availability becomes the main driver of REE distribution at higher exposure levels, while biological control is more important at lower concentrations. Similar patterns have been observed in aquatic systems, where higher REE levels lead to a clearer preservation of environmental geochemical signatures in organisms [59].

In contrast in *P. clarkii*, the smooth and coherent decrease from LREEs to HREEs, combined with Ce and Eu anomalies close to unity, indicates that REE distributions closely reflect environmental conditions rather than strong biological fractionation [60]. This pattern is consistent with the general geochemical behavior of REEs in aquatic systems, where fractionation is largely controlled by complexation and particle reactivity, resulting in characteristic LREE enrichment and relatively smooth normalized profiles [61]. The low variability observed suggests that *P. clarkii* integrates environmental REE signals with limited biological discrimination.

Overall, some taxa (*e.g., S. glanis*) exhibit species-specific modulation of REE patterns, reflecting physiological regulation and ecological traits, particularly under low exposure conditions, whereas others (*e.g., P. clarkii*) preserve environmental REE signatures with minimal biological fractionation [56].

### 4.2. Organic Contaminants: PAHs, NDL-PCBs and Pesticides

PAHs and NDL-PCBs are anthropogenic persistent organic contaminants widely distributed in both aquatic and terrestrial ecosystems. PAHs mainly originate from the combustion of fossil fuels and the release of petroleum products, and are associated with potentially carcinogenic, mutagenic, and endocrine-disrupting effects [62]. NDL-PCBs, formerly used in industrial applications such as dielectric fluids, plasticizers, adhesives, and flame retardants, persist in the environment despite the ban on their production and exert toxic effects by interfering with calcium homeostasis, inhibiting specific enzymatic systems, and altering neurotransmission [63].

Pesticides, widely used in agriculture to protect crops, include both persistent and bioaccumulative compounds as well as more degradable substances; even at low concentrations, they may induce physiological alterations, oxidative stress, and cellular damage [64].

The investigation of these three classes of organic contaminants was essential, as *S. glanis* and *P. clarkii* are capable of bioaccumulating them, and the assessment of their levels was crucial to ensure their safe use in animal feed and human food.

In the present study, PAH concentrations were below the LOQ in both species, with chrysene representing the only detectable compound at very low levels (0.9 µg/kg). These findings contrast with those of Squadrone et al. [65], who reported substantially higher ΣPAH concentrations (26.90 ± 49.50 µg/kg) in *S. glanis* from the Po River basin (Northern Italy), indicating a greater contamination pressure in that area. Similarly, Liu et al. [66] documented higher ΣPAH levels (4.32 ± 0.0710 µg/kg) in *P. clarkii* from Hubei (Central China), further supporting the comparatively low PAH burden observed in the present study.

Although no specific regulatory limits for PAHs in animal feed have been established, the concentrations detected here are well below the maximum levels set for human consumption under Regulation (EU) 2023/915 [35].

Similarly, NDL-PCBs were also below the LOQ in both species. In contrast, substantially higher ΣNDL-PCB levels have been reported in *S. glanis* from the Po River basin (135.6 µg/kg) [63] and from Lakes of Mantova (21.9 µg/kg) [67], again suggesting a higher level of environmental contamination compared to the present study sites. To the best of our knowledge, no studies have specifically investigated the occurrence of NDL-PCBs in *P. clarkii*, highlighting a current gap in the literature.

The values for both IAS were below the regulatory limits for animal feed established by Regulation (EU) No 277/2012 [68], as well as with those for human consumption defined by Regulation (EU) 2023/915 [35].

Pesticide residues in *S. glanis* from both Great Lake and Small Lake, as well as in *P. clarkii* from Great Lake, were below the limit of quantification (LOQ: 0.002–0.005 µg/kg). In contrast, Luo et al. [69] reported mean pesticide residues of 0.72 µg/kg in *P. clarkii* from rice-crayfish co-culture systems in Huai’an, China. Similarly, Zeinab and Kather [70] found pesticide residues ranging from non-detectable to 6875 µg/kg in *P. clarkii* from Muweis Canal, Zagazig, Egypt, and from ND to 9653 µg/kg in *Clarias gariepinus* (African sharptooth catfish).

The residues observed in the present study remain well below the maximum limits established for both animal feed and human consumption by Regulation (EC) No 396/2005 [71].

Overall, the concentrations of PAHs, NDL-PCBs, and pesticide residues in *S. glanis* and *P. clarkii* were below LOQ and EU regulatory thresholds. These findings suggest a low organic contamination pressure at the study sites and a limited risk for food and feed safety.

### 4.3. Microplastics

MPs, defined as plastic fragments ranging from 1 µm to 5 mm, are increasingly recognized as emerging pollutants posing potential risks to environmental, animal, and human health. Based on their origin, MPs are classified as primary or secondary. Primary MPs are intentionally manufactured for use in products such as cosmetics, paints, detergents, and fertilizers, whereas secondary MPs originate from the fragmentation of larger plastic items through physical and chemical processes, including ultraviolet (UV) photodegradation and mechanical abrasion [72,73].

Due to their widespread distribution in aquatic and terrestrial ecosystems, monitoring MPs in sentinel species such as *S. glanis* and *P. clarkii* is essential. In the present study, all intestinal tracts analyzed from *S. glanis* (Great and Small Lake) and *P. clarkii* (Great Lake) were contaminated with MPs, confirming their pervasive presence [74].

A previous study demonstrated that *S. glanis* can ingest plastic debris, with plastics detected in stomach contents [75], highlighting the species’ susceptibility to plastic exposure. MP concentrations observed in the intestinal tracts of *S. glanis* from Great Lake and Small Lake (4.2 ± 2.15 MPs per intestinal tract and 4.4 ± 2.70 MPs per intestinal tract, respectively) are consistent with values reported in fish from East India, where an average of 3.0 ± 1.8 MPs per gastrointestinal tract was recorded across 45 individuals belonging to eight different species [76]. In contrast, the concentrations observed in this study are substantially higher than those reported for *S. glanis* in the Amir–Kalayeh Wetland (Northern Iran), where MP levels were approximately 0.25 MPs per intestinal tract [77]. This marked discrepancy may reflect differences in local pollution sources, varying degrees of anthropogenic pressure, or distinct environmental conditions across the studied regions.

The predominant MP colors in *S. glanis* were black (47.6%) > white (35.7%) > blue (16.7%) in Great Lake, whereas in Small Lake blue (40.9%) > black (27.3%) > white (22.8%), with minor contributions from green and orange (4.5%). These findings are consistent with those of Patidar et al. [76], who also reported blue, black, and white as the most common colors, with smaller proportions of green and orange. Similarly, Nejat et al. [77] found that blue and transparent MPs were the dominant types in *S. glanis* specimens.

From a chemical perspective, the predominant polymers were PE > PET, PP in Great Lake and PP > PA, PET in Small Lake. Similar polymer compositions have been reported by Patidar et al. [76], whereas Nejat et al. [77] identified PA as the dominant polymer in *S. glanis*.

A positive correlation between fish weight and MP concentration was observed for *S. glanis* in both lakes, suggesting that larger individuals may accumulate more MPs, potentially due to longer exposure times or differences in feeding behavior. This contrasts with other studies reporting either no correlation [77] or negative [76], indicating that the relationship between organism size and MP ingestion is complex and may depend on ecological and environmental factors.

Finally, the PHI classification indicated that PA falls within hazard category III, PE and PET within category II, and PP within category I, in agreement with the work of Patidar et al. [76]. This suggests a progressively decreasing potential ecological risk from PA to PP, as higher PHI categories are generally associated with greater environmental persistence, higher likelihood of ingestion by aquatic organisms, and increased potential for toxicological effects due to fragmentation and bioavailability [31,32]. The mean MPs length was 252 µm Great Lake and 250 Small Lake, indicating the predominance of small-sized MPs within the studied samples. This size range is environmentally relevant, as MPs below approximately 300 µm are readily ingestible by a wide range of aquatic organisms, thereby increasing their potential for trophic transfer [78].

Regarding *P. clarkii*, the MP concentrations observed in this study (2.7 ± 2.39 MPs per intestinal tract) were higher than those reported in individuals from Lake Candia (0.7 MPs per intestinal tract) and from Jianli, Hubei Province, China (0.71 ± 0.18 MPs per individual) [79,80]. It should be noted that these comparisons are intended solely to report concentration ranges observed in the same species across different studies, rather than to infer ecological differences among study areas, which may be influenced by distinct environmental conditions.

In terms of color distribution, blue, black, and white were the dominant categories, while the main polymer types were PET > PE > PP. This pattern is comparable to that reported by Pastorino et al. [79] for *P. clarkii* from Lake Candia, although PE was not detected in that study. Comparable results were also reported by Zhang et al. [80], who identified blue and transparent MPs with PP:PE and PE as the main polymer types.

No correlation between body weight and MP concentration was observed in *P. clarkii* in the present study, whereas Pastorino et al. [79] reported a negative relationship, further highlighting the variability of MP ingestion dynamics among populations and environmental contexts.

The PHI values for the polymers identified in *P. clarkii* ranged from low (PP) to medium (PE and PET). To date, no previous studies have assessed the PHI of MPs in *P. clarkii*, highlighting the novelty of this aspect of the present study. Although no high-hazard polymers were detected, the occurrence of PE and PET suggests a moderate potential ecological risk, as these polymers are characterized by greater environmental persistence and potential toxicological impacts compared to PP [30,31]. Similarly to the findings observed in catfish, the MPs detected in *P. clarkii* showed a mean size of approximately 294 µm, indicating the predominance of small-sized MPs that are more readily ingestible by aquatic organisms, potentially increasing trophic transfer and ecological exposure [78].

These results support the role of *S. glanis* and *P. clarkii* as effective bioindicators of MP pollution, as MPs were detected in water of the Lakes of Avigliana [40]. Furthermore, the color distribution observed in this study aligns with patterns reported in European freshwater systems, where black (33.4%), blue (31.5%), and white (27.2%) are the most prevalent colors. These are commonly associated with anthropogenic sources such as synthetic textile fibers, vehicle-related emissions, and the fragmentation of plastic debris, indicating a strong human influence in the study area [81]. Similarly, the polymer composition identified (PE, PP, PET, and PA) reflects global production trends, with PE (34.1%), PP (22.9%), and PET (17%) among the most widely produced polymers worldwide, largely used in packaging, textiles, and consumer products [81], while PA is extensively used in fishing gear (*e.g.,* nets, lines, and ropes) due to its durability and flexibility, representing a likely source of contamination in aquatic environments [82].

Concerning the potential use of *S. glanis* and *P. clarkii* as alternative feed and food sources, no specific regulatory limits have yet been established for the presence of MPs in feed or food. As MPs are still considered emerging pollutants, their implications for animal and human health cannot be fully assessed, and their safety cannot be confirmed in the absence of specific regulatory thresholds and risk assessment frameworks. At the European level, the only regulation currently in force (Regulation (EU) 2023/2055) [83] restricts the intentional addition of MPs in products such as cosmetics, detergents, and fertilizers, but does not address their occurrence in food or feed.

### 4.4. Multivariate Analysis

The multivariate analysis (NMDS), based on trace elements, REEs, and MP concentrations, corroborated the patterns observed in the univariate analyses, highlighting clear spatial differentiation between Great Lake and Small Lake. In particular, *S. glanis* samples from the two lakes were positioned at opposite ends of the NMDS1 axis, indicating a marked separation in their contaminant profiles. Consistent with these findings, the univariate analyses of *S. glanis* individuals showed that trace elements (20 out of 21) and REEs (12 out of 16) exhibited significantly higher concentrations in Small Lake, further emphasizing the differences in contamination between the two sites. Moreover, these results are supported by environmental reports [40], which have also documented differences in contamination levels between the lakes, revealing higher contamination in Small Lake. Taken together, these findings support the potential use of *S. glanis* as a bioindicator, although confirmation with simultaneous water, sediment, and trophic-web data would strengthen this interpretation.

The revised PERMANOVA analysis further supported these patterns, revealing significant site-related differences in elemental profiles of *S. glanis* between Great Lake and Small Lake. Furthermore, PERMDISP analyses indicates that the observed PERMANOVA differences were primarily associated with shifts in multivariate centroid positions rather than heterogeneous within-group variability. Conversely, spatial comparisons for *P. clarkii* were not feasible due to its absence in Small Lake [19].

Additionally, the NMDS analysis revealed a clear separation between the two IAS, *S. glanis* and *P. clarkii*, from Great Lake, particularly along the NMDS2 axis. This pattern suggests species–specific differences in contaminant accumulation. Such differences are consistent with the variability observed in the concentrations of individual contaminants and can be explained by the distinct ecological and biological traits of the two species.

*S. glanis* is a large, long-lived, top predator with higher trophic level feeding habits, which may promote biomagnification of certain contaminants [84]. In contrast, *P. clarkii* is a smaller, benthic, omnivorous species with different feeding strategies, habitat use, and metabolic rates [85], all of which can influence contaminant uptake and bioaccumulation.

This multivariate approach represents a relatively novel contribution to studies investigating these IAS as bioindicators. To the best of our knowledge, the available literature remains limited and has predominantly focused on single contaminants [43,44,45,46,49,52,63,65,66,67,69,70,76,77,79,80], rather than adopting an integrated, multi-contaminant framework combined with multivariate analyses. Therefore, our results provide new insights into the combined occurrence patterns and spatial distribution patterns of multiple contaminants in IAS.

## 5. Conclusions

*S. glanis* and *P. clarkii* are highly invasive alien species that threaten freshwater biodiversity, making their management a priority. This study shows that, beyond their ecological impact, these species can serve as effective bioindicators of environmental contamination in the Avigliana Lakes. Both species accumulated trace elements and REEs; *S. glanis* showed higher concentrations in Small Lake, reflecting greater local pollution. Organic contaminants (PAHs, NDL-PCBs, and pesticides) were largely absent, while MPs were detected in all individuals, confirming their ubiquitous presence in freshwater ecosystems. Results from univariate analyses were corroborated by multivariate analyses, revealing clear differences between sites and species.

From a feed and food perspective, most regulated contaminants were below applicable EU thresholds (except Pb in *S. glanis* from Small Lake that exceeded the maximum level established for fish muscle intended for human consumption) providing preliminary contaminant-related information relevant to evaluating possible future use of these species as alternative resources for animals and humans. However, REEs and MPs remain unregulated, highlighting critical gaps in risk assessment frameworks, which are still insufficient to fully evaluate their potential environmental and health risks.

Future studies should expand the analysis to additional contaminants (including Hg and As speciation) and other IAS to better understand the risks and opportunities associated with their use as bioindicators and alternative feed or food resources. Moreover, before using these IAS as alternative feed and food, it is essential to broaden knowledge on their nutritional value, consumer exposure, toxicological risks, processing effects, bioavailability, seasonal variability, and full compliance with food and feed regulations.

Overall, these findings highlight the dual role of *S. glanis* and *P. clarkii* as both ecological threats and valuable tools for environmental monitoring and sustainable resource utilization.

## Data Availability

The original contributions presented in this study are included in the article/Supplementary Material. Further inquiries can be directed to the corresponding authors.

## Supplementary Materials

The following supporting information can be downloaded at: https://www.mdpi.com/article/10.3390/jox16030109/s1, Text S1. Additional information on Materials and Methods for trace elements and rare earth elements (certified reference material used; recoveries; precision/RSD; blank values; internal standards). Text S2. Detailed PHI calculation procedure. Figure S1. Chondrite-normalized rare earth element (REE) patterns for *P. clarkii* from Great Lake, *S. glanis* from Great Lake, and *S. glanis* from Small Lake, shown from left to right. Table S1. Comparison of trace element concentrations expressed in mg/kg w.w. in *Silurus glanis* collected from Avigliana Great Lake and Avigliana Small Lake using the Kruskal–Wallis test with Bonferroni correction. For each trace element, the sample size (N), mean ± standard deviation (SD), median, interquartile range (IQR), minimum–maximum range, *p*-value, and effect size are reported. Values below LOD (0.0003 mg/kg) were treated as equal to LOD. Table S2. Trace element concentrations expressed in mg/kg w.w. in *Procambarus clarkii* collected from Avigliana Great Lake. For each trace element, the sample size (N), mean ± standard deviation (SD), median, interquartile range (IQR) and minimum–maximum range are reported. Values below LOD (0.0003 mg/kg) were treated as equal to LOD. Table S3. Comparison of rare earth element (REE) concentrations expressed in mg/kg w.w. in *Silurus glanis* collected from Avigliana Great Lake and Avigliana Small Lake using the Kruskal–Wallis test with Bonferroni correction. For each REE, the sample size (N), mean ± standard deviation (SD), median, interquartile range (IQR), minimum–maximum range, *p*-value, and effect size are reported. Values below LOD (0.0003 mg/kg) were treated as equal to LOD. Table S4. Rare earth element (REE) concentrations expressed in mg/kg w.w. in *Procambarus clarkii* collected from Avigliana Great Lake. For each REE, the sample size (N), mean ± standard deviation (SD), median, interquartile range (IQR), and minimum–maximum range are reported. Values below LOD (0.0003 mg/kg) were treated as equal to LOD. Table S5. Comparison of MP concentrations per intestinal tract in *Silurus glanis* collected from Avigliana Great Lake and Avigliana Small Lake using the Kruskal–Wallis test with Bonferroni correction. The sample size (N), mean ± standard deviation (SD), median, interquartile range (IQR), minimum–maximum range, *p*-value, and effect size are reported. Table S6. MP concentrations per intestinal tract in *Procambarus clarkii* collected from Avigliana Great Lake. The sample size (N), mean ± standard deviation (SD), median, interquartile range (IQR), and minimum–maximum range are reported.

## Abbreviations

The following abbreviations are used in this manuscript:

GC–MS	Gas Chromatography–Mass Spectrometry
IAS	Invasive Alien Species
ICP-MS	Inductively Coupled Plasma Mass Spectrometry
HPLC	High-Performance Liquid Chromatography
HREEs	Heavy Rare Earth Elements
LOQ	Limit Of Quantification
LREEs	Low Rare Earth Elements
MPs	Microplastics
NDL-PCBs	Non-Dioxin-Like Polychlorinated Biphenyls
PA	Polyamide
PAHs	Polycyclic Aromatic Hydrocarbons
PE	Polyethylene
PET	Polyethylene terephthalate
PHI	Polymer Hazard Index
PP	Polypropylene
PVC	Polyvinyl Chloride
REEs	Rare Earth Elements

## Appendix A

### List of Investigated Pesticides

The pesticides investigated (N = 95) were as follows: 2-phenylphenol, acrinathrin, aldrin, azoxystrobin, bifenthrin, bixafen, boscalid, bromopropylate, bromuconazole, cadusafos, cis-chlordane, trans-chlordane, chlorfenvinphos, chlorpropham, chlorpyrifos, chlorpyrifos-methyl, cyfluthrin, λ-cyhalothrin, cypermethrin, cyprodinil, p,p′-DDD, o,p′-DDT, p,p′-DDE, p,p′-DDT, cis-deltamethrin, diazinon, dieldrin, difenoconazole, α-endosulfan, β-endosulfan, endosulfan sulfate, endrin, endrin ketone, EPN, esfenvalerate, ethion, etofenprox, famoxadone, fenarimol, fenazaquin, fenitrothion, fenpropathrin, fenpropimorph, fenthion, fenvalerate, fipronil, fipronil sulfone, flucythrinate, fludioxonil, fluquinconazole, τ-fluvalinate, α-HCH, β-HCH, heptachlor, heptachlor endo-epoxide (isomer A, trans), heptachlor exo-epoxide (isomer B, cis), iprodione, isocarbophos, isoprothiolane, kresoxim-methyl, lindane (γ-HCH), malaoxon, malathion, mepanipyrim, methacrifos, metalaxyl, methoxychlor, metolachlor, oxadixyl, oxychlordane, paraoxon-methyl, parathion, parathion-methyl, pendimethalin, permethrin, phenthoate, phosalone, phosmet, piperonyl butoxide, pirimiphos, pirimiphos-methyl, procymidone, profenofos, propargite, pyridaben, pyrimethanil, resmethrin, spiromesifen, tebufenpyrad, tefluthrin, tetramethrin, tolclofos-methyl, triadimefon, trifluralin, and vinclozolin. Triphenylphosphine (TPP) was used as the internal standard.
