# Supplementary material for: From Ecological Threats to Monitoring Tools: Multi-Contaminant Profiles in Silurus glanis and Procambarus clarkii for Pollution Tracking and Preliminary Food/Feed Safety Assessment
Source: J Xenobiot. 2026 Jun 9;16(3):109. doi: 10.3390/jox16030109 (PMC13302379; doi:10.3390/jox16030109)
Supplement: Supplementary file 1 [file jox-16-00109-s001.zip › jox-4321823 new SM.pdf]

Supplementary Materials: From Ecological Threats to Monitoring Tools: Multi-Contaminant Profiles in *Silurus glanis* and *Procambarus clarkii* for Pollution Tracking and Preliminary Food/Feed Safety Assessment

Sara Glorio Patrucco[1], Roberta Giugliano[2], Alessandra Griglione[1], Giorgia Zicarelli[1], Camilla Mossotto[1], Leo Costa[1], Giuseppe Esposito[1], Alice Gabetti[1], Serena Anselmi[3], Tecla Bentivoglio[3], Barbara Vivaldi[2], Valentina Ciccotelli[2], Bruno Aimone[4], Marino Prearo[1], Damià Barceló[5], Monia Renzi[6], Stefania Squadrone[1] and Paolo Pastorino[1,*]

Text S1. Additional information on Materials and Methods for trace elements and rare earth elements (certified reference material used; recoveries; precision/RSD; blank values; internal standards).

Certified Reference Material includes: BCR-668 (Mussel Tissue) and NIST 1566b (Oyster Tissue)
Mean % recovery (R%) and precision (RSD%) calculated on BCR-668 (Mussel Tissue)

	BCR -668		
Element		R%	RSD%
Y	93.4	6.3	
La	91.9	5.1	
Ce	100.3	5.6	
Pr	104.2	4.6	
Nd	100.1	4.3	
Sm	107.3	5.2	
Eu	111.7	4.3	
Gd	106.8	4.8	
Tb	110.4	3.9	
Dy	99.8	4.5	
Er	101.3	5.1	
U	99.6	4.7	
	Oyster Tissue		
Element		R%	RSD%

Al	113.2	8.4
As	107.8	5.4
Cd	101.7	4.6
Co	99.6	4.2
Cu	96.4	5.4
Fe	103.3	5.7
Pb	105.9	6.7
Mn	96.7	5.3
Ni	98.7	4.8
Rb	93.6	4.2
Se	109.4	8.6
Ag	92.8	6.8
V	100.2	5.6
Zn	104.6	5.3

Blank values

Element	Concentration (mg/kg)
Sc	0.0008
Y	0.0008
Ce	0.0009
Gd	0.0009
La	0.0005
Lu	0.0003
Sm	0.0009
Dy	0.0009
Er	0.0009
Eu	0.0004
Ho	0.0003
Nd	0.0004
Pr	0.0009
Tb	0.0002
Tm	0.0002
Yb	0.0009

Element	Concentration (mg/kg)
Ag	0.0018
Al	2.26
As	0.048
Be	0.00
Cd	0.0017
Co	0.0016
Cr	0.018
Cs	0.0004
Cu	0.18
Fe	0.26
Ga	0.0005
In	0.0012
Mg	5.18
Mn	0.65
Mo	0.0109
Ni	0.22
Rb	0.0051
Se	0.0000
Sr	0.0650
V	0.007
Zn	0.18

Internal Standard: Rh and Ge at 10 µg/mL

Text S2. Detailed PHI calculation procedure**.**

The Polymer Hazard Index (PHI) was calculated for each species and sampling site according to the following equation:

$$PHI = \Sigma (Pn \times Sn) \text{ (Eq. S1)}$$

where Pn is the relative proportion of each polymer type in the sample and Sn is the hazard score assigned to each polymer type based on Lithner et al., [30] classification (hazard scores: PET = 4, PE = 11, PP = 1, PA = 50).

The relative proportion (Pn) was calculated as:

$$Pn = ni / N \text{ (Eq. S2)}$$

where ni is the number of MPs of a given polymer type and N is the total number of particles identified in each sample.
The hazard levels were given according to the classification of Ranjani et al., [31]:

- PHI = 0-1 → Hazard Category I (risk category minor);
- PHI = 1-10 → Hazard Category II (risk category medium);
- PHI = 10-100 → Hazard Category III (risk category high).

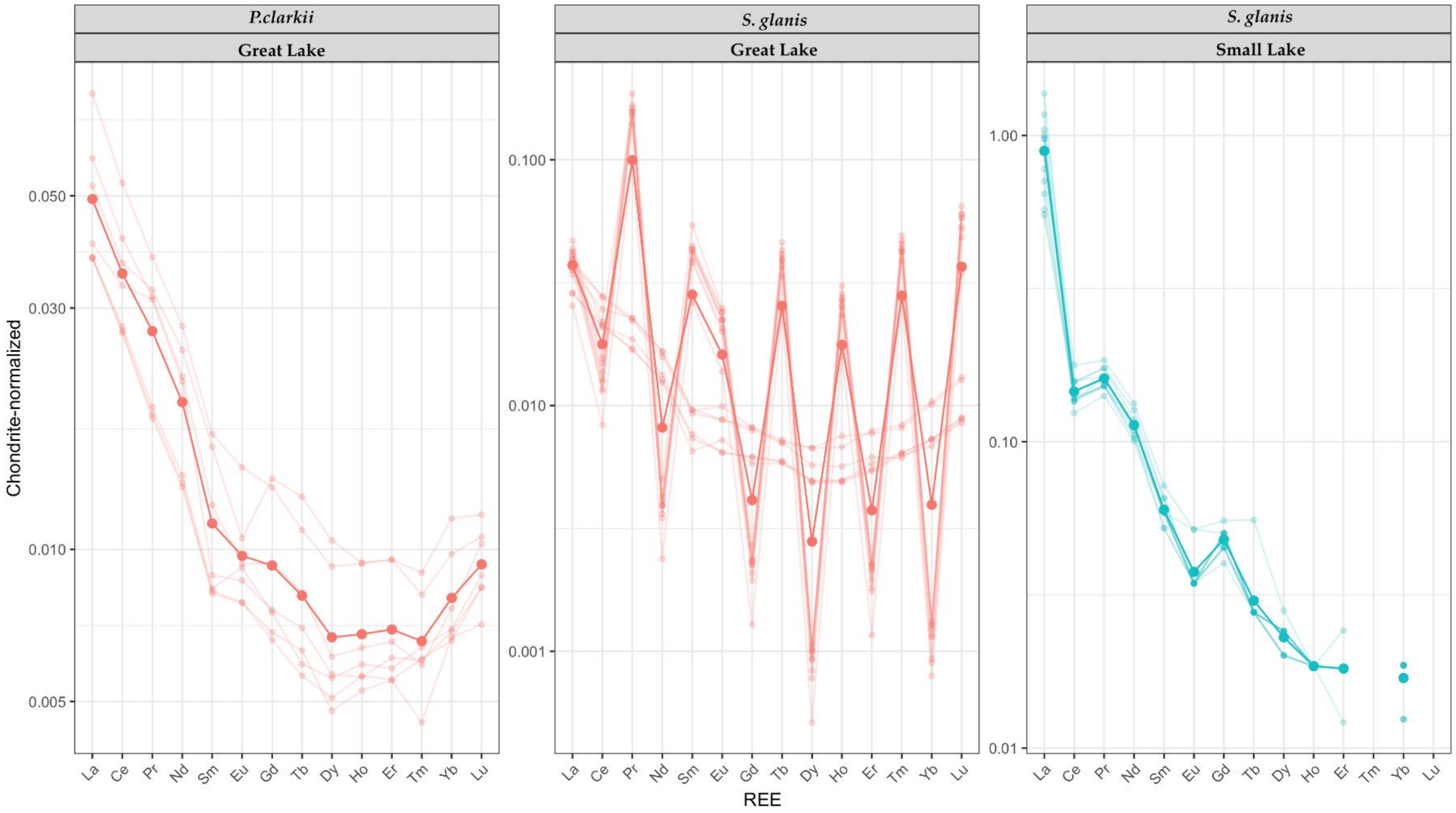

Figure S1. Chondrite-normalized rare earth element (REE) patterns for *P. clarkii* from Great Lake, *S. glanis* from Great Lake, and *S. glanis* from Small Lake, shown from left to right.

Table S1. Comparison of trace element concentrations expressed in mg/kg w. w. in *Silurus glanis* collected from Avigliana Great Lake and Avigliana Small Lake using the Kruskal–Wallis test with Bonferroni correction. For each trace element, the sample size (N), mean ± standard deviation (SD), median, interquartile range (IQR), minimum–maximum range, p-value, and effect size are reported. Values below LOD (0.0003 mg/kg) were treated as equal to LOD.

Trace Element	Lake	N	Mean ± SD	Median	IQR	Min–Max	P-value	Effect Size
Ag	Great Lake	15	0.00070 ± 0.000239	0.00059	0.00027	0.00038 - 0.00117	0.0000181802	0.841
Ag	Small Lake	15	0.01182 ± 0.002562	0.01100	0.00400	0.00900 - 0.01600		
As	Great Lake	15	0.01728 ± 0.009625	0.01343	0.00797	0.00677 - 0.03760	0.0000185434	0.840
As	Small Lake	15	0.19409 ± 0.147721	0.12800	0.05400	0.10000 - 0.52200		
Ba	Great Lake	15	0.82737 ± 0.297068	0.81058	0.33473	0.37439 - 1.32870	0.0000185434	0.840
Ba	Small Lake	15	7.24991 ± 1.04646	7.09900	1.22500	6.09000 - 9.53600		
Bi	Great Lake	15	0.00435 ± 0.002067	0.00385	0.00137	0.00219 - 0.01002	0.0000185434	0.840
Bi	Small Lake	15	0.03318 ± 0.015587	0.03200	0.01300	0.01700 - 0.07200		
Cd	Great Lake	15	0.00311 ± 0.001502	0.00237	0.00134	0.00191 - 0.00749	0.0000184825	0.840
Cd	Small Lake	15	0.03036 ± 0.006697	0.02900	0.01100	0.02100 - 0.03900		
Co	Great Lake	15	0.00662 ± 0.002547	0.00583	0.00218	0.00460 - 0.01489	0.0000185434	0.840
Co	Small Lake	15	0.12636 ± 0.036590	0.12200	0.03300	0.08900 - 0.22000		
Cr	Great Lake	15	0.26389 ± 0.645956	0.06760	0.05313	0.03552 - 2.58214	0.0001363789	0.748
Cr	Small Lake	15	1.62491 ± 1.094762	1.44500	0.45650	0.59200 - 4.46700		
Cs	Great Lake	15	0.01649 ± 0.002431	0.01667	0.00249	0.00999 - 0.01953	0.0000184217	0.840
Cs	Small Lake	15	0.12191 ± 0.030817	0.10900	0.04700	0.08300 - 0.18200		
Cu	Great Lake	15	4.52911 ± 3.781702	3.74608	0.84997	2.53319 - 18.08048	0.0000185434	0.8340
Cu	Small Lake	15	50.59218 ± 4.662585	49.94200	3.04700	44.04800 - 62.11500		
Ga	Great Lake	15	0.02174 ± 0.004617	0.02153	0.00486	0.01297 - 0.03143	0.0001086050	0.759
Ga	Small Lake	15	0.00955 ± 0.005165	0.00700	0.00500	0.00400 - 0.01900		
In	Great Lake	15	0.00030 ± 0.000005	0.00030	0	0.00030 - 0.00032	0.0000035900	0.914
In	Small Lake	15	0.01964 ± 0.003355	0.02000	0.00500	0.01400 - 0.02400		
Mn	Great Lake	15	0.72782 ± 0.130374	0.76532	0.18475	0.47086 - 0.91315	0.0000185434	0.840
Mn	Small Lake	15	15.15855 ± 1.392002	15.23000	1.51800	13.31700 - 18.27000		
Mo	Great Lake	15	0.09026 ± 0.295885	0.01439	0.00380	0.00843 - 1.15975	0.0002060514	0.728
Mo	Small Lake	15	0.129273 ± 0.078013	0.105000	0.023500	0.074000 - 0.356000		
Ni	Great Lake	15	0.62896 ± 1.984515	0.11634	0.03418	0.07427 - 7.80091	0.0002065686	0.728
Ni	Small Lake	15	1.63100 ± 0.470357	1.35900	0.52100	1.21400 - 2.79400		
Pb	Great Lake	15	0.16249 ± 0.024137	0.16607	0.02851	0.10123 - 0.19444	0.0000185434	0.840

Pb	Small Lake	15	1.85755 ± 0.266456	1.86200	0.27000	1.42200 - 2.23700		
Rb	Great Lake	15	1.64087 ± 0.550222	1.63861	0.87309	0.96207 - 2.61434	0.0000185434	0.840
Rb	Small Lake	15	43.11800 ± 4.752350	41.72000	7.38850	37.54900 - 50.91200		
Se	Great Lake	15	0.18542 ± 0.042921	0.17654	0.05441	0.11554 - 0.25767	0.0000185434	0.840
Se	Small Lake	15	5.15936 ± 1.174069	5.19500	1.18550	3.46400 - 7.80000		
Sr	Great Lake	15	3.53264 ± 0.679847	3.61711	1.08980	2.28083 - 4.52574	0.0000249384	0.843
Sr	Small Lake	15	52.87582 ± 5.056317	53.34500	4.92850	45.47900 - 63.13900		
Tl	Great Lake	15	0.00306 ± 0.001929	0.00230	0.00113	0.00145 - 0.00875	0.0004503917	0.688
Tl	Small Lake	15	0.00654 ± 0.002067	0.00600	0.00300	0.00400 - 0.01000		
U	Great Lake	15	0.01551 ± 0.003390	0.01548	0.00421	0.01054 - 0.02420	0.0000185434	0.840
U	Small Lake	15	0.14200 ± 0.018612	0.14200	0.02900	0.11900 - 0.17000		
V	Great Lake	15	0.00983 ± 0.001991	0.00925	0.00181	0.00597 - 0.01474	0.0000184825	0.840
V	Small Lake	15	0.15682 ± 0.02678	0.15200	0.04600	0.13100 - 0.20000		

Table S2. Trace element concentrations expressed in mg/kg w. w. in *Procambarus clarkii* collected from Avigliana Great Lake. For each trace element, the sample size (N), mean ± standard deviation (SD), median, interquartile range (IQR) and minimum–maximum range are reported. Values below LOD (0.0003 mg/kg) were treated as equal to LOD.

Trace Element	Lake	N	Mean ± SD	Median	IQR	Min–Max
Ag	Great Lake	40	0.01660 ± 0.003951	0.01568	0.00450	0.01222 - 0.02328
As	Great Lake	40	0.24505 ± 0.023470	0.23967	0.02469	0.20944 - 0.28202
Ba	Great Lake	40	1.16765 ± 0.339045	1.23592	0.41047	0.66439 - 1.66890
Bi	Great Lake	40	0.00744 ± 0.005186	0.00524	0.00213	0.00434 - 0.01878
Cd	Great Lake	40	0.00587 ± 0.002338	0.00626	0.00349	0.00313 - 0.00905
Co	Great Lake	40	0.02305 ± 0.013775	0.01648	0.01336	0.01199 - 0.05058
Cr	Great Lake	40	0.74834 ± 1.590685	0.12981	0.11701	0.08357 - 4.35271
Cs	Great Lake	40	0.01823 ± 0.002568	0.01801	0.00061	0.01458 - 0.02328
Cu	Great Lake	40	16.94713 ± 3.146496	15.37056	3.35923	14.24701- 22.70140
Ga	Great Lake	40	0.02145 ± 0.001780	0.02139	0.00095	0.01783 - 0.02354
In	Great Lake	40	0.00031 ± 0.000035	0.00030	0	0.00030 - 0.00039
Mn	Great Lake	40	3.15608 ± 1.363718	2.28336	2.01178	2.05987 - 5.09483
Mo	Great Lake	40	0.37794 ± 0.904178	0.02919	0.03009	0.02349 - 2.42792
Ni	Great Lake	40	0.62465 ± 0.461708	0.55947	0.31129	0.27339 - 1.60414
Pb	Great Lake	40	0.21263 ± 0.030390	0.22146	0.02717	0.15922 - 0.25589
Rb	Great Lake	40	2.31895 ± 0.400836	2.27317	0.36511	1.92463 - 3.10968

Se	Great Lake	40	0.20377 ± 0.072063	0.17330	0.06009	0.12040 - 0.33548
Sr	Great Lake	40	13.52844 ± 9.838705	10.63937	11.78030	4.62467 - 29.79474
Tl	Great Lake	40	0.00401 ± 0.002137	0.00320	0.00102	0.00171 - 0.00842
U	Great Lake	40	0.02207 ± 0.005721	0.01843	0.00797	0.01756 - 0.03147
V	Great Lake	40	0.02696 ± 0.011923	0.02176	0.01492	0.01591 - 0.04405

Table S3. Comparison of rare earth element (REE) concentrations expressed in mg/kg w. w. in *Silurus glanis* collected from Avigliana Great Lake and Avigliana Small Lake using the Kruskal–Wallis test with Bonferroni correction. For each REE, the sample size (N), mean ± standard deviation (SD), median, interquartile range (IQR), minimum–maximum range, p-value, and effect size are reported. Values below LOD (0.0003 mg/kg) were treated as equal to LOD.

REE	Lake	N	Mean ± SD	Median	IQR	Min–Max	P-value	Effect Size
Ce	Great Lake	15	0.01088 ± 0.003822	0.00955	0.00553	0.00511- 0.01712	0.0000183611	0.840
Ce	Small Lake	15	0.08945 ± 0.009267	0.08500	0.01250	0.07600 - 0.10900		
Dy	Great Lake	15	0.00070 ± 0.000576	0.00030	0.00093	0.00030 - 0.00167	0.0000104391	0.864
Dy	Small Lake	15	0.00573 ± 0.000647	0.00600	0.00100	0.00500 - 0.00700		
Er	Great Lake	15	0.00062 ± 0.000390	0.00037	0.00063	0.00030 - 0.00131	0.0000114374	0.861
Er	Small Lake	15	0.00300 ± 0.000447	0.00300	0	0.00200 - 0.00400		
Eu	Great Lake	15	0.00093 ± 0.000430	0.00116	0.00079	0.00037 - 0.00144	0.0000122465	0.858
Eu	Small Lake	15	0.00218 ± 0.000404	0.00200	0	0.00200 - 0.00300		
Gd	Great Lake	15	0.00082 ± 0.000515	0.00047	0.00078	0.00030 - 0.00162	0.0000167838	0.844
Gd	Small Lake	15	0.00955 ± 0.000820	0.01000	0.00100	0.00800 - 0.01100		
Ho	Great Lake	15	0.00096 ± 0.000560	0.00126	0.00111	0.00030 - 0.00166	0.7661757568	0.058
Ho	Small Lake	15	0.00100 ± 0	0.00100	0	0.00100 - 0.00100		
La	Great Lake	15	0.00871 ± 0.001400	0.00907	0.00148	0.00597 - 0.01096	0.0000185434	0.840
La	Small Lake	15	0.20836 ± 0.061109	0.22800	0.08050	0.12900 - 0.32100		
Lu	Great Lake	15	0.00090 ± 0.000535	0.00116	0.00109	0.00030 - 0.00155	0.0024822552	0.593
Lu	Small Lake	15	0.00030 ± 0	0.00030	0	0.00030 - 0.00030		
Nd	Great Lake	15	0.00372 ± 0.002550	0.00196	0.00416	0.00109 - 0.00763	0.0000184217	0.840
Nd	Small Lake	15	0.05182 ± 0.004513	0.05100	0.00400	0.04600 - 0.06100		
Pr	Great Lake	15	0.00916 ± 0.006412	0.01285	0.01240	0.00155 - 0.01712	0.0270064468	0.434
Pr	Small Lake	15	0.01482 ± 0.001168	0.01500	0.00150	0.01300 - 0.01700		
Sc	Great Lake	15	0.00319 ± 0.003742	0.00030	0.00636	0.00030 - 0.00940	0.0022832674	0.598
Sc	Small Lake	15	0.00836 ± 0.002580	0.00900	0.00250	0.00400 - 0.01200		
Sm	Great Lake	15	0.00432 ± 0.002713	0.00585	0.00513	0.00100 - 0.00829	0.0000261518	0.825
Sm	Small Lake	15	0.00918 ± 0.000874	0.00900	0.00050	0.00800 - 0.01100		

Tb	Great Lake	15	0.00094 ± 0.000569	0.00121	0.00112	0.00030 - 0.00166	0.8930649440	0.026
Tb	Small Lake	15	0.00109 ± 0.000302	0.00100	0	0.00100 - 0.00200		
Tm	Great Lake	15	0.00075 ± 0.000399	0.00097	0.00078	0.00030 - 0.00123	0.0024822552	0.593
Tm	Small Lake	15	0.00030 ± 0	0.00030	0	0.00030 - 0.00030		
Y	Great Lake	15	0.00847 ± 0.001575	0.00831	0.00106	0.00505 - 0.01133	0.0000184825	0.840
Y	Small Lake	15	0.03700 ± 0.003606	0.03800	0.00500	0.03100 - 0.04200		
Yb	Great Lake	15	0.00071 ± 0.000539	0.00030	0.00087	0.00030 - 0.00167	0.0000088871	0.871
Yb	Small Lake	15	0.00273 ± 0.000467	0.00300	0.00050	0.00200 - 0.00300		

Table S4. Rare earth element (REE) concentrations expressed in mg/kg w.w. in *Procambarus clarkii* collected from Avigliana Great Lake. For each REE, the sample size (N), mean ± standard deviation (SD), median, interquartile range (IQR), and minimum–maximum range are reported. Values below LOD (0.0003 mg/kg) were treated as equal to LOD.

REE	Lake	N	Mean ± SD	Median	IQR	Min–Max
Ce	Great Lake	40	0.02152 ± 0.005892	0.02039	0.00717	0.01640 - 0.03248
Dy	Great Lake	40	0.00167 ± 0.000550	0.00141	0.00059	0.00119 - 0.00259
Er	Great Lake	40	0.00115 ± 0.000299	0.00101	0.00039	0.00091 - 0.00158
Eu	Great Lake	40	0.00056 ± 0.000135	0.00053	0.00010	0.00046 - 0.00084
Gd	Great Lake	40	0.00185 ± 0.000604	0.00151	0.00083	0.00132 - 0.00275
Ho	Great Lake	40	0.00036 ± 0.000101	0.00030	0.00010	0.00030 - 0.00051
La	Great Lake	40	0.01153 ± 0.003726	0.00941	0.00424	0.00880 - 0.01866
Lu	Great Lake	40	0.00030 ± 0	0.00030	0	0.00030 - 0.00030
Nd	Great Lake	40	0.00893 ± 0.002697	0.00982	0.00440	0.00608 - 0.01264
Pr	Great Lake	40	0.00249 ± 0.000751	0.00287	0.00122	0.00167 - 0.00348
Sc	Great Lake	40	0.00792 ± 0.001169	0.00729	0.00115	0.00705 - 0.00978
Sm	Great Lake	40	0.00172 ± 0.000583	0.00136	0.00088	0.00125 - 0.00258
Tb	Great Lake	40	0.00034 ± 0.000064	0.00030	0.00005	0.00030 - 0.00046
Tm	Great Lake	40	0.00030 ± 0	0.00030	0	0.00030- 0.00030
Y	Great Lake	40	0.01172 ± 0.003148	0.01029	0.00349	0.00942 - 0.01675
Yb	Great Lake	40	0.00129 ± 0.000306	0.00112	0.00031	0.00106 - 0.00185

Table S5. Comparison of MP concentrations per intestinal tract in *Silurus glanis* collected from Avigliana Great Lake and Avigliana Small Lake using the Kruskal–Wallis test with Bonferroni correction. The sample size (N), mean ± standard deviation (SD), median, interquartile range (IQR), minimum–maximum range, p-value, and effect size are reported.

	Lake	N	Mean ± SD	Median	IQR	Min–Max	P-value	Effect Size
**MPs/intestinal tract**	Great Lake	15	4.2 ± 2.15	5.5	4.0	1- 6	0.8989	0.032

**MPs/intestinal tract**	Small Lake	15	4.4 ± 2.70	4.0	3.0	1 - 8

Table S6. MP concentrations per intestinal tract in *Procambarus clarkii* collected from Avigliana Great Lake. The sample size (N), mean ± standard deviation (SD), median, interquartile range (IQR), and minimum–maximum range are reported.

	Lake	N	Mean ± SD	Median	IQR	Min–Max
**MPs/intestinal tract**	Great Lake	40	2.7 ± 2.39	2.5	5.0	1 - 6

References

30. Lithner, D.; Larsson, Å.; Dave, G. Environmental and Health Hazard Ranking and Assessment of Plastic Polymers Based on Chemical Composition. *Science of The Total Environment* **2011**, *409*, 3309–3324, doi:10.1016/j.scitotenv.2011.04.038.
31. Ranjani, M.; Veerasingam, S.; Venkatachalapathy, R.; Mugilarasan, M.; Bagaev, A.; Mukhanov, V.; Vethamony, P. Assessment of Potential Ecological Risk of Microplastics in the Coastal Sediments of India: A Meta-Analysis. *Marine Pollution Bulletin* **2021**, *163*, 111969, doi:10.1016/j.marpolbul.2021.111969.
